# Reframing Alzheimer’s Disease Through a Redox-Metabolic Framework

**DOI:** 10.3390/ijms27188294

**Published:** 2026-09-17

**Authors:** Amador Velázquez De Castro-Bono, Gracia Castro-Luna, José Luis Guil-Guerrero

**Affiliations:** 1 Hospital Central de La Defensa “Gómez Ulla”, 28047 Madrid, Spain; avelazquezcb@gmail.com; 2 Department of Nursing, Physiotherapy and Medicine, University of Almería, 04120 Almería, Spain; graciacl@ual.es; 3 Food Technology Division, University of Almería, 04120 Almería, Spain

**Keywords:** Alzheimer’s disease, oxidative stress, redox biology, mitochondrial dysfunction, glucose hypometabolism, neuroinflammation, ferroptosis, cuproptosis, metal dyshomeostasis, NRF2, precision medicine, metabolomics

## Abstract

Alzheimer’s disease (AD) has been conceptualised as a proteinopathy driven by amyloid-β plaques and hyperphosphorylated tau neurofibrillary tangles. AD should not be understood as exclusively a proteinopathy or a metabolic/redox disorder, but as a network of interacting processes in which metabolic dysfunction, mitochondrial impairment, redox dysregulation, amyloid-β, tau, neuroinflammation, metal dyshomeostasis, and regulated cell death reinforce one another. This review examines AD through a redox-metabolic framework integrating cerebral glucose metabolism, insulin signalling, mitochondrial bioenergetics, metal homeostasis, and regulated cell death. We discuss how glucose hypometabolism, impaired oxidative phosphorylation, and weakened antioxidant defences may promote reactive oxygen species production and self-reinforcing oxidative and metabolic dysfunction. We examine interactions with amyloid-β and tau pathology, glial immunometabolism, gut–brain signalling, neuroinflammation, and metal-mediated toxicity. Advances in multi-omics, blood-based metabolomic and lipidomic biomarkers, and imaging may enable earlier biological stratification. Therapeutic strategies targeting mitochondria, NRF2 signalling, metabolic dysfunction, metal dyshomeostasis, and regulated oxidative cell death are evaluated based on current evidence and their potential complementarity with amyloid-directed therapies. Although temporal relationships remain unresolved, redox-metabolic dysfunction may represent an early determinant and amplifier of neuronal vulnerability. Evidence for lecanemab and donanemab supports an integrative rather than replacement model of AD treatment. This framework may facilitate earlier diagnosis, patient stratification, and complementary disease-modifying interventions.

## 1. Introduction: Reframing Alzheimer’s Disease Through a Redox-Metabolic Framework

For over three decades, research on Alzheimer’s disease (AD) has largely been shaped by the amyloid cascade hypothesis. According to this model, the aggregation of amyloid beta (Aβ) peptides and the associated formation of tau pathology kick off a cascade of events that cause synapse dysfunction, neuronal death, and ultimately cognitive deterioration [1]. Although numerous amyloid-directed approaches have failed to demonstrate clinical benefit, recent phase 3 trials have shown that selected anti-amyloid therapies can modestly slow clinical decline in early symptomatic AD. These findings reinforce the therapeutic relevance of amyloid pathology while also highlighting the need to investigate complementary mechanisms that may influence disease progression and treatment response [2].

In addition, it is now known, due to progress made in neuroimaging, metabolomics, redox biology, and mitochondrial neuroscience, that metabolic and redox disturbances can arise several years or even decades before the development of cognitive symptoms [3]. These findings are consistent with the mitochondrial cascade hypothesis, which proposes that age-associated mitochondrial disturbances may contribute to susceptibility to neurodegeneration [4,5]. These developments have led to a reconsideration of AD as a complex, multifactorial systems disorder, comprising intricate interrelationships between energy metabolism disturbances, redox dysregulation, inflammation, protein aggregation, and age-related loss of cellular robustness.

In this evolving model, there is growing interest in the mutual interaction between metabolism and oxidative stress. The brain’s high metabolic demand requires maintaining an intricate balance between energy metabolism and ROS production. Disruption of this balance may contribute to mitochondrial dysfunction, lipid peroxidation, altered signalling, and inflammatory responses. All of which create a conducive environment for the onset and enhancement of amyloid and tau pathologies [6,7].

Rather than supplanting established models of amyloid-β and tau pathology, the redox-metabolic framework considered here places these pathological processes within a broader systems-level model of neurodegeneration. Protein aggregation, neuroinflammation, mitochondrial dysfunction, metabolic impairment, and oxidative stress may influence one another through interconnected feedback mechanisms. Thus, the framework does not require a single initiating event; instead, it proposes that early metabolic and redox disturbances may increase neuronal vulnerability and subsequently interact with amyloid-β and tau pathology to reinforce disease progression [6,7].

### 1.1. From Downstream Oxidative Damage to Upstream Redox Dysregulation

Oxidative stress has long been established as a key characteristic of the AD brain. Early neuropathological studies showed that levels of oxidised lipids, proteins, and nucleic acids increase in vulnerable brain structures. At the time, these changes were commonly accepted as a secondary effect of neuronal degeneration, not the driving force of AD [6,8]. In the traditional free radical theory of ageing, ROS were considered passive products formed in the late phases of cellular deterioration [9].

This understanding shaped treatment approaches, and many therapies used nonspecific antioxidants, such as vitamins E and C. Even though some epidemiological evidence showed the positive effects of antioxidant treatment, the randomised clinical studies revealed little benefit from them, so the role of oxidative stress in AD became questionable [8].

Over the past two decades, however, major advances in redox biology have fundamentally altered this perspective. ROS are now recognised as potentially damaging molecules, and also as essential signalling mediators involved in synaptic plasticity, long-term potentiation, vascular regulation, immune responses, and adaptive cellular stress signalling [10]. Under physiological conditions, tightly controlled ROS production serves critical homeostatic functions throughout the nervous system [11].

Problems arise when ROS generation chronically exceeds the capacity of endogenous antioxidant systems. Experimental and clinical evidence suggests that age-related mitochondrial dysfunction may contribute to progressive increases in oxidative burden long before the appearance of overt dementia [5]. Impairments in the electron transport chain increase electron leakage and superoxide production, while concurrent reductions in antioxidant defences—including dysregulation of the nuclear factor erythroid 2-related factor 2 (NRF2)-glutathione axis—limit the brain’s capacity to restore redox equilibrium [12].

Sustained redox imbalance could affect several pathogenic pathways associated with AD development. ROS production could cause lipid peroxidation, disrupt mitochondrial energetics, alter calcium metabolism, affect the integrity of nucleic acids, and mediate inflammatory signals [13]. At the same time, oxidative modifications of lipids and proteins could contribute to ferroptosis susceptibility, while oxidation of mitochondria-associated molecular patterns, such as mitochondrial DNA, could initiate an innate immune response via NLRP3 inflammasome activation [14,15].

Thus, these data support considering redox dysregulation as an active component of the pathological network that may influence disease progression, while its temporal relationship with amyloid, tau, metabolic dysfunction, and neuroinflammation remains unresolved. In addition, oxidative stress likely contributes to a two-way interaction with amyloid and tau pathology, forming vicious circles promoting neuronal deterioration [6,7].

### 1.2. Metabolic Resilience and Vulnerability of the Ageing Brain

The concept of metabolic resilience offers an effective approach to explain the growing vulnerability of the ageing brain to the development of neurodegenerative disorders. Despite the fact that the brain accounts for only 2% of the total body weight, it utilises about 20% of the oxygen and glucose required by the whole body in a resting state, which demonstrates the extreme energy requirements of the processes involved in neural functioning [2].

Metabolic resilience is the ability of neural circuits to sustain normal performance under energy stress. Healthy brains possess considerable metabolic flexibility and can use alternative energy substrates, such as lactate or ketone bodies, when glucose availability is reduced [2]. At the same time, cellular adaptation mechanisms, including antioxidant signalling through the NRF2 pathway, help maintain redox homeostasis under stress [10,16].

Ageing progressively reduces this adaptive capacity. One important contributor is brain insulin resistance, a state in which neurons and glia become less responsive to insulin signalling through the PI3K/Akt pathway despite normal or elevated insulin levels; because insulin receptors are densely expressed in the hippocampus and cortex and normally support glucose uptake, synaptic plasticity, and neuronal survival, their desensitisation directly impairs these functions, a phenomenon frequently described as a “Type 3 diabetes” phenotype, although this terminology remains debated and is not universally accepted. Impaired insulin signalling reduces glucose utilisation efficiency and has been associated with alterations in GLUT1- and GLUT3-mediated transport, particularly within regions that exhibit early vulnerability to AD pathology [17].

Functional neuroimaging studies have consistently demonstrated regional glucose hypometabolism in the precuneus, posterior cingulate cortex, and temporoparietal regions years before clinical diagnosis [18]. Earlier FDG-PET investigations similarly demonstrated that cerebral glucose hypometabolism may precede detectable cognitive impairment and predict future disease progression [3].

As energetic stress persists, compensatory mechanisms become increasingly strained. Reduced metabolic flexibility, impaired mitochondrial function, and diminished antioxidant capacity collectively increase susceptibility to oxidative damage and inflammatory activation [4,7]. Rather than a sudden catastrophic event, neurodegeneration may therefore emerge through a gradual erosion of metabolic resilience that compromises the ability of neurons and glia to maintain homeostasis in the face of cumulative biological stressors.

This perspective helps explain why ageing remains the strongest risk factor for AD. Progressive declines in bioenergetic efficiency, mitochondrial integrity, and redox regulation create a particular physiological environment. Within this environment, multiple pathogenic processes—including amyloid accumulation, tau pathology, neuroinflammation, and ferroptotic vulnerability—can interact and amplify one another [5,6].

**Figure 1:**
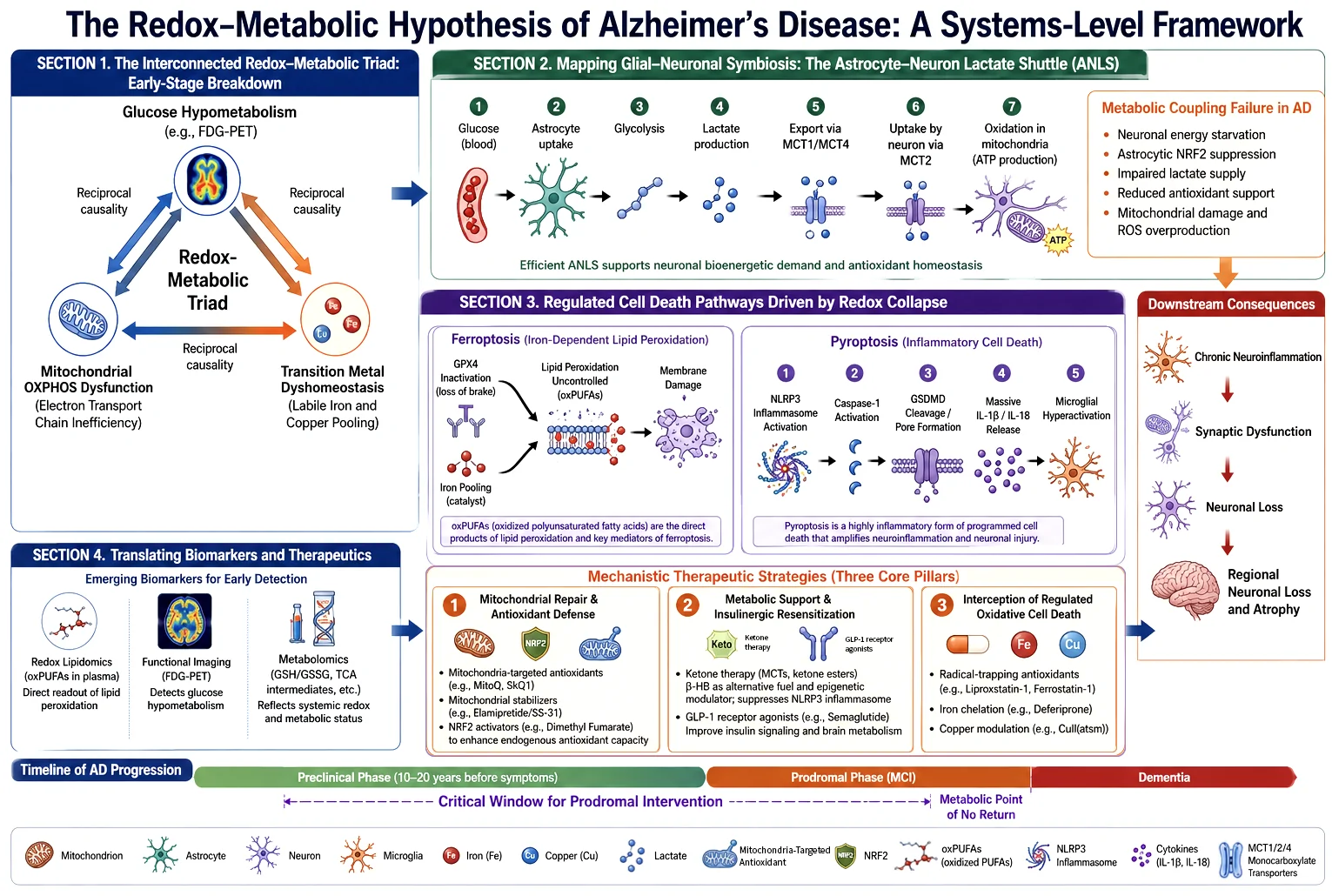
The Redox-Metabolic Framework of Alzheimer’s Disease: A Systems-Level Model. This schematic integrates the principal metabolic and redox-related processes considered in this review, including glucose hypometabolism, mitochondrial OXPHOS dysfunction, redox dysregulation, transition-metal dyshomeostasis, impaired astrocyte–neuron metabolic coupling, neuroinflammatory signalling, and regulated cell death. The figure is organised along a conceptual disease-progression axis, illustrating how metabolic and redox disturbances may interact and become amplified across preclinical, prodromal, and dementia stages. The directional arrows represent proposed mechanistic relationships and downstream consequences rather than a definitive or obligatory causal sequence, as temporal relationships among individual processes may vary by disease stage, cell type, and biological context. The lower panels highlight emerging multi-omics and imaging approaches for early biological detection and biomarker-guided stratification, together with three investigational therapeutic domains targeting mitochondrial and redox function, metabolic resilience, and regulated cell death. The figure therefore represents a systems-level framework rather than a validated linear disease pathway or therapeutic algorithm; the optimal timing and clinical efficacy of redox-metabolic interventions remain to be established.

Figure 1 summarises the redox-metabolic pathway described in this review, showing the suggested progression from age-dependent loss of metabolic resilience to the development of neurodegenerative pathology. Rather than presenting Alzheimer’s disease as a set of individual events occurring one after another, the redox-metabolic pathway emphasises interconnections among glucose hypometabolism, mitochondrial dysfunction, transition metal dyshomeostasis, oxidative stress, neuroinflammation, and cell death that contribute to neurodegeneration. It is proposed that the described processes create self-propelling biological networks that gradually disrupt the neuronal system and lead to the onset of cognitive impairment.

The figure also highlights potential opportunities for early diagnosis and intervention through biomarker-guided precision medicine approaches.

## 2. Physiological Redox and Metabolic Coupling in the Healthy Brain

Before examining the metabolic and oxidative disturbances associated with Alzheimer’s disease, it is essential to understand the physiological mechanisms that maintain redox homeostasis in the healthy brain. Neuronal function depends on a highly coordinated interplay among mitochondrial energy production, glial-neuronal metabolic coupling, antioxidant defence systems, and tightly regulated reactive oxygen and nitrogen species (ROS/RNS) signalling. Although ROS and RNS are frequently associated with oxidative damage, contemporary redox biology recognises that they also serve as important signalling mediators involved in synaptic plasticity, neurovascular regulation, immune responses, and adaptive cellular stress signalling [8,19]. The integrity of these interconnected systems underpins the metabolic resilience required to sustain the brain’s exceptionally high energetic demands throughout life.

### 2.1. Compartmentalisation of ROS/RNS in Neuronal Signalling and Synaptic Plasticity

ROS and RNS have traditionally been viewed as potentially harmful byproducts of aerobic metabolism. However, accumulating evidence demonstrates that these molecules also function as tightly regulated signalling mediators that contribute to neuronal communication, synaptic plasticity, vascular regulation, and adaptive stress responses [19]. Consequently, their biological effects depend less on absolute concentration than on precise spatial localisation, temporal regulation, and cellular context.

In the healthy brain, highly reactive species such as superoxide (O_2_•^−^) exhibit limited diffusion distances and short half-lives, restricting their actions to local microdomains [20]. This compartmentalisation allows ROS-mediated signalling to occur without provoking widespread oxidative damage. During periods of increased synaptic activity, activation of N-methyl-D-aspartate (NMDA) receptors promotes transient calcium influx and localised ROS generation, particularly through NADPH oxidase complexes [21]. Rather than inducing cellular injury, these physiological ROS bursts contribute to long-term potentiation (LTP), a fundamental mechanism underlying learning and memory [19].

ROS comprise chemically distinct species with different reactivities, lifetimes, and cellular distributions. Superoxide is relatively short-lived and exhibits limited diffusion, whereas hydrogen peroxide (H_2_O_2_) is longer-lived and can participate in regulated redox signalling and, under appropriate conditions, diffuse between cellular compartments [22]. Hydroxyl radicals (•OH) are highly reactive and largely non-selective, whereas lipid peroxyl radicals propagate chain reactions within PUFA-containing biological membranes. Consequently, statements referring generically to “ROS” should be interpreted according to the specific reactive species and compartment involved [22].

In this review, “oxidative stress” refers to a disturbance in the balance between oxidant production and antioxidant capacity that alters redox homeostasis; “redox dysregulation” denotes broader disruption of regulated redox signalling and control; “oxidative damage” refers to molecular injury caused by reactive species; and “ROS overproduction” refers specifically to increased generation of ROS beyond the range required for physiological signalling. These terms are related but are not interchangeable.

Reactive nitrogen species participate in analogous signalling networks. Nitric oxide (NO), for example, regulates neuronal function through reversible post-translational modifications such as S-nitrosylation, thereby influencing protein activity, synaptic transmission, and neuronal survival [23]. Through these highly controlled mechanisms, ROS and RNS link cellular metabolism to synaptic adaptation and cognitive function.

Importantly, the distinction between physiological signalling and pathological oxidative stress is quantitative, not absolute. Redox homeostasis depends on maintaining ROS/RNS production within a range that supports cellular signalling while avoiding cumulative molecular damage. Failure of this balance reflects a critical early step in the transition from physiological adaptation to neurodegeneration [19].

### 2.2. The Astrocyte-Neuron Lactate Shuttle and Glial-Neuronal Metabolic Coupling

The human brain presents a unique bioenergetic challenge. Although neurons account for much of the brain’s energy consumption, they possess limited glycolytic capacity and rely heavily on oxidative metabolism to sustain synaptic activity. This apparent paradox is partially resolved through close metabolic cooperation between neurons and astrocytes, a relationship commonly described by the Astrocyte-Neuron Lactate Shuttle (ANLS) hypothesis [24].

Astrocytes act as metabolic intermediaries between the cerebral vasculature and neuronal networks. Through specialised perivascular endfeet, astrocytes take up glucose via GLUT1 transporters and preferentially metabolise it through aerobic glycolysis. During periods of intense synaptic activity, astrocytic glycolysis supports glutamate uptake from the synaptic cleft while simultaneously generating lactate, which is exported through monocarboxylate transporters (MCT1 and MCT4). Neurons subsequently import lactate through the high-affinity transporter MCT2 and utilise it as an oxidative substrate for ATP generation [25].

Beyond substrate transfer, this metabolic partnership contributes to cellular redox regulation. Conversion of pyruvate to lactate in astrocytes regenerates cytosolic NAD^+^ required to sustain glycolytic flux, whereas neuronal lactate oxidation generates reducing equivalents that support mitochondrial oxidative phosphorylation [26]. Consequently, glial-neuronal metabolic coupling links energy production with the maintenance of intracellular redox balance.

This metabolic segregation is also associated with distinct redox phenotypes. Neurons prioritise efficient ATP production through tightly organised mitochondrial respiratory supercomplexes, whereas astrocytes exhibit greater antioxidant capacity and maintain higher basal glutathione levels [27]. Astrocytic metabolism therefore serves both bioenergetic and neuroprotective functions, supporting neuronal survival under conditions of physiological stress.

Although the ANLS remains one of the most influential models of cerebral energy metabolism, its quantitative contribution to neuronal bioenergetics remains debated. Alternative models suggest that neurons may utilise glucose to a greater extent than originally proposed and that the relative importance of lactate varies by brain region, physiological state, and experimental methodology [28]. Nevertheless, substantial evidence supports a central role for astrocyte-neuron metabolic cooperation in maintaining cerebral energy homeostasis and redox resilience [25].

### 2.3. Antioxidant Defence Systems: Glutathione, Thioredoxin, and NRF2 Signalling

Maintaining physiological redox signalling requires a well-established antioxidant network that limits excessive ROS accumulation while preserving essential signalling functions. In the brain, this defence system is organised around three interconnected components: the glutathione (GSH) system, the thioredoxin/peroxiredoxin (Trx/Prx) pathway, and the NRF2 transcriptional programme [13].

Glutathione represents the principal intracellular redox buffer and plays a central role in detoxifying reactive oxygen species. Through the action of glutathione peroxidases, GSH reduces hydrogen peroxide and lipid hydroperoxides while limiting oxidative damage to proteins, lipids, and nucleic acids [16]. Astrocytes possess significantly greater glutathione reserves than neurons and continuously support neuronal antioxidant defences through metabolic cooperation and precursor supply [29].

Complementing this system, the thioredoxin/peroxiredoxin pathway functions as a major protein repair and peroxide detoxification network. Driven by NADPH-dependent reduction reactions, thioredoxin restores oxidised protein thiols, while peroxiredoxins remove hydrogen peroxide and organic peroxides generated during normal cellular metabolism [16].

The master regulator coordinating these antioxidant defences is nuclear factor erythroid 2-related factor 2 (NRF2). Under basal conditions, NRF2 is retained in the cytoplasm through association with Kelch-like ECH-associated protein 1 (KEAP1), which targets NRF2 for proteasomal degradation [30]. In response to oxidative or electrophilic stress, modification of KEAP1 cysteine residues stabilises NRF2 and promotes its nuclear translocation, where it activates antioxidant response element (ARE)-dependent transcription of genes involved in glutathione synthesis, thioredoxin recycling, NADPH production, and cellular detoxification [10,29].

NRF2 activity exhibits marked cellular asymmetry within the central nervous system. Mature neurons express substantially lower NRF2 levels than astrocytes, rendering them more dependent on glial antioxidant support [27,31]. Consequently, astrocytic NRF2 activation plays a disproportionately important role in maintaining neuronal redox homeostasis and protecting vulnerable neural circuits from oxidative stress.

**Figure 2:**
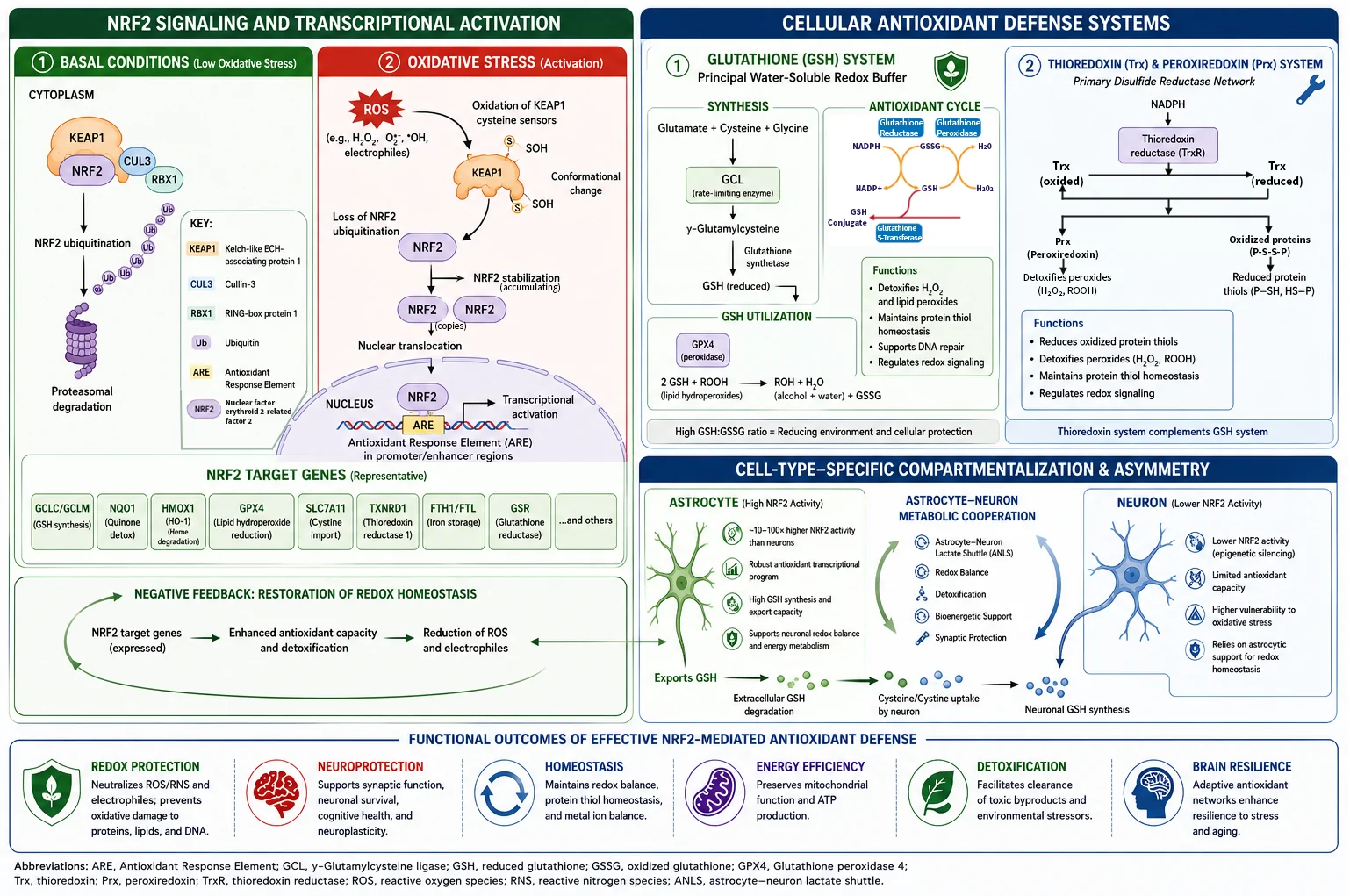
NRF2 Signalling, Antioxidant Defence Systems and Astrocyte–Neuron Asymmetry in the Healthy Brain. This schematic summarises the molecular machinery that maintains redox homeostasis under physiological conditions. Under basal conditions, KEAP1 promotes NRF2 ubiquitination and proteasomal degradation. Oxidants such as H_2_O_2_ or electrophiles modify KEAP1 cysteine sensors, stabilising NRF2, which translocates to the nucleus and induces ARE-dependent genes involved in glutathione synthesis, detoxification, thioredoxin recycling, and iron handling, with feedback restoring redox balance. The glutathione and thioredoxin/peroxiredoxin systems buffer peroxides and preserve protein thiol status while permitting redox signalling. NRF2 activity is higher in astrocytes than in neurons, and astrocyte-derived glutathione precursors may support neuronal defences, alongside the proposed astrocyte–neuron lactate shuttle. Compartmentalised ROS/RNS signalling, mitochondrial energy metabolism and NADPH-generating pathways are described in the text and not depicted.

Figure 2 summarises the physiological integration of redox signalling, glial-neuronal metabolic cooperation, and antioxidant defence systems. Together, compartmentalised ROS/RNS signalling, astrocyte-neuron lactate exchange, mitochondrial oxidative phosphorylation, and NRF2-dependent antioxidant pathways form a coordinated metabolic network that supports neuronal function while preserving redox homeostasis. These mechanisms collectively establish the biological foundation for metabolic resilience and provide a framework for understanding how their disruption may contribute to neurodegenerative disease.

## 3. Metabolic and Mitochondrial Dysfunction in Alzheimer’s Disease

AD is increasingly recognised as a disorder characterised not only by protein aggregation but also by progressive disturbances in cerebral energy metabolism. Converging evidence from multiple lines of research from neuroimaging, metabolomics, and mitochondrial biology indicates that alterations in glucose utilisation, insulin signalling, mitochondrial function, and redox homeostasis emerge during the prodromal stages of disease, often preceding overt cognitive impairment [3,17,18,32]. These abnormalities may reduce neuronal resilience and interact with amyloid-beta (Aβ), tau pathology, and neuroinflammation through complex bidirectional mechanisms. Consequently, metabolic dysfunction is increasingly viewed as an important component of the broader pathogenic network underlying AD rather than a simple downstream consequence of neurodegeneration. Broader syntheses of mitochondrial dysfunction in AD, spanning bioenergetic failure, impaired mitophagy, and disrupted mitochondrial dynamics, reinforce this view [33,34].

### 3.1. Glucose Hypometabolism, Insulin Resistance, and the Prodromal Timeline

One of the most reproducible biological findings in AD is regional cerebral glucose hypometabolism. Functional neuroimaging studies using fluorodeoxyglucose positron emission tomography (FDG-PET) consistently demonstrate reduced glucose utilisation in the posterior cingulate cortex, precuneus, and temporoparietal regions years before the onset of clinically detectable dementia [18]. Earlier longitudinal FDG-PET studies similarly showed that cerebral glucose hypometabolism may precede measurable cognitive decline and predict future disease progression [3].

In this context, metabolic abnormalities often emerge before extensive amyloid deposition and may occur partially independently of classical proteinopathic changes. These observations have led investigators to propose that metabolic dysfunction may represent an early indicator of disease vulnerability rather than merely a secondary consequence of neurodegeneration [3].

Metabolomic analyses provide additional support for this concept. Alterations in circulating amino acids, carbohydrates, and lipid intermediates have been identified during preclinical and prodromal stages of AD, suggesting that systemic metabolic changes accompany the earliest phases of disease development. Although no single metabolomic signature has yet achieved diagnostic specificity, these findings reinforce the concept that disturbances in energy metabolism emerge early in the pathogenic process [35].

A growing body of evidence also implicates impaired insulin signalling in AD. Brain insulin resistance has frequently been described as a “Type 3 diabetes” phenotype, although the terminology remains debated and is not universally accepted [17]. Beyond its role in glucose metabolism, insulin signalling contributes to synaptic plasticity, neuronal survival, neurotransmitter regulation, and cellular bioenergetics [36]. Dysregulation of the PI3K/Akt pathway may impair astrocytic glycolysis, alter glial-neuronal metabolic coupling, and reduce the metabolic support required to sustain synaptic activity during periods of increased neuronal demand [17]. This specific mechanistic link is drawn primarily from experimental models rather than direct human evidence [12].

Taken together, these findings suggest that impaired glucose utilisation and insulin signalling may contribute to a gradual erosion of metabolic resilience that precedes overt neurodegeneration.

### 3.2. Mitochondrial Bioenergetic Failure, Oxidative Phosphorylation Deficits, and Reactive Oxygen Species Production

Mitochondria occupy a central position at the intersection of cellular metabolism and redox regulation. The Mitochondrial Cascade Hypothesis proposes that age-related mitochondrial dysfunction may represent an important upstream contributor to AD pathogenesis [4,5]. Although this hypothesis remains influential, it is best viewed as complementary to rather than competitive with amyloid- and tau-centred models, as increasing evidence supports reciprocal interactions among mitochondrial dysfunction, protein aggregation, neuroinflammation, and oxidative stress [37].

Structural and functional mitochondrial abnormalities are consistently observed in AD. Neuronal mitochondria exhibit altered morphology characterised by increased fragmentation, disrupted cristae architecture (which destabilises respiratory chain supercomplexes), and imbalances in mitochondrial fission and fusion dynamics [38,39]. These alterations can compromise mitochondrial bioenergetic efficiency and neuronal capacity to meet high energy demands [32]. However, mitochondrial dysfunction should not be equated automatically with increased ROS production, as ROS generation depends on the respiratory-chain site, mitochondrial redox state, membrane potential, substrate availability, and electron-transfer conditions. At the biochemical level, deficits in oxidative phosphorylation (OXPHOS) have been reported across multiple studies [40]. Reduced activity of electron transport chain complexes, particularly Complex I (NADH oxidoreductase) and Complex IV (cytochrome c oxidase), contributes to diminished ATP generation and impaired mitochondrial respiration [40]. Mitochondrial ROS generation is particularly associated with specific sites within the respiratory chain, principally Complexes I and III. At Complex I, both forward electron transfer and reverse electron transfer (RET) can favour superoxide formation under particular energetic and redox conditions, whereas Complex III can generate superoxide through reactions involving semiquinone intermediates [41]. By contrast, Complex IV primarily catalyses the four-electron reduction of O_2_ to H_2_O and is not generally regarded as a major site of electron-leak-derived superoxide. Thus, impaired electron transport does not necessarily produce ROS simply because electron flow is reduced; rather, alterations in electron-transfer kinetics and the mitochondrial energetic state may redistribute ROS generation and modify its magnitude and intracellular localisation [42].

The reactive species generated by mitochondria are chemically heterogeneous and exert different biological effects. Superoxide (O_2_•^−^) is relatively short-lived and has limited ability to cross biological membranes, whereas hydrogen peroxide (H_2_O_2_), generated through spontaneous or superoxide-dismutase-catalysed dismutation, is longer-lived and can participate in regulated redox signalling as well as oxidative damage when present in excess [13]. Hydroxyl radicals (•OH) are highly reactive and largely non-selective, while lipid peroxyl radicals can propagate chain reactions within PUFA-containing membranes. Consequently, the term “ROS” encompasses species with markedly different reactivities, lifetimes, and cellular distributions and should not be interpreted as a single biochemical entity [43].

Increased generation of reactive species, when sustained beyond the capacity of antioxidant and repair systems, can disrupt neuronal homeostasis. Elevated oxidative stress may promote lipid peroxidation, protein oxidation, mitochondrial DNA damage, calcium dysregulation, and activation of cellular stress-response pathways [10]. These effects depend on the identity, concentration, and intracellular localisation of the reactive species, as well as on the capacity of antioxidant and repair systems. At the same time, age-related reductions in antioxidant defences—including impaired NRF2-dependent signalling and glutathione metabolism—may limit neurons’ capacity to maintain redox homeostasis under sustained metabolic stress [31]. Accordingly, redox dysregulation should be distinguished from ROS overproduction: the former encompasses broader disturbances in redox control and signalling, whereas the latter refers specifically to increased generation of reactive oxygen species beyond physiological requirements.

Mitochondrial dysfunction may also contribute to chronic neuroinflammation. Damaged mitochondria can release damage-associated molecular patterns (DAMPs), including mitochondrial DNA and other mitochondrial constituents, which can activate innate immune pathways such as the NLRP3 inflammasome [14]. Mitochondrial impairment can therefore provide a mechanistic connection between altered bioenergetics, redox imbalance, and inflammatory signalling, although the directionality of these interactions is not necessarily unidirectional. Neuroinflammatory processes can impair mitochondrial function, while mitochondrial ROS and DAMP release may amplify inflammatory signalling. This reciprocal interaction may establish a self-reinforcing network linking bioenergetic dysfunction, redox dysregulation, and immune activation [6,7].

Overall, mitochondrial abnormalities in AD are best understood as components of an interconnected metabolic and redox network rather than as evidence of a single upstream mitochondrial defect. Alterations in OXPHOS, electron-transfer kinetics, mitochondrial dynamics, antioxidant capacity, calcium handling, and inflammatory signalling may interact to increase neuronal energetic vulnerability. This framework is consistent with the broader redox-metabolic model of AD while avoiding the assumption that mitochondrial dysfunction necessarily precedes amyloid or tau pathology or represents the sole initiating event.

**Figure 3:**
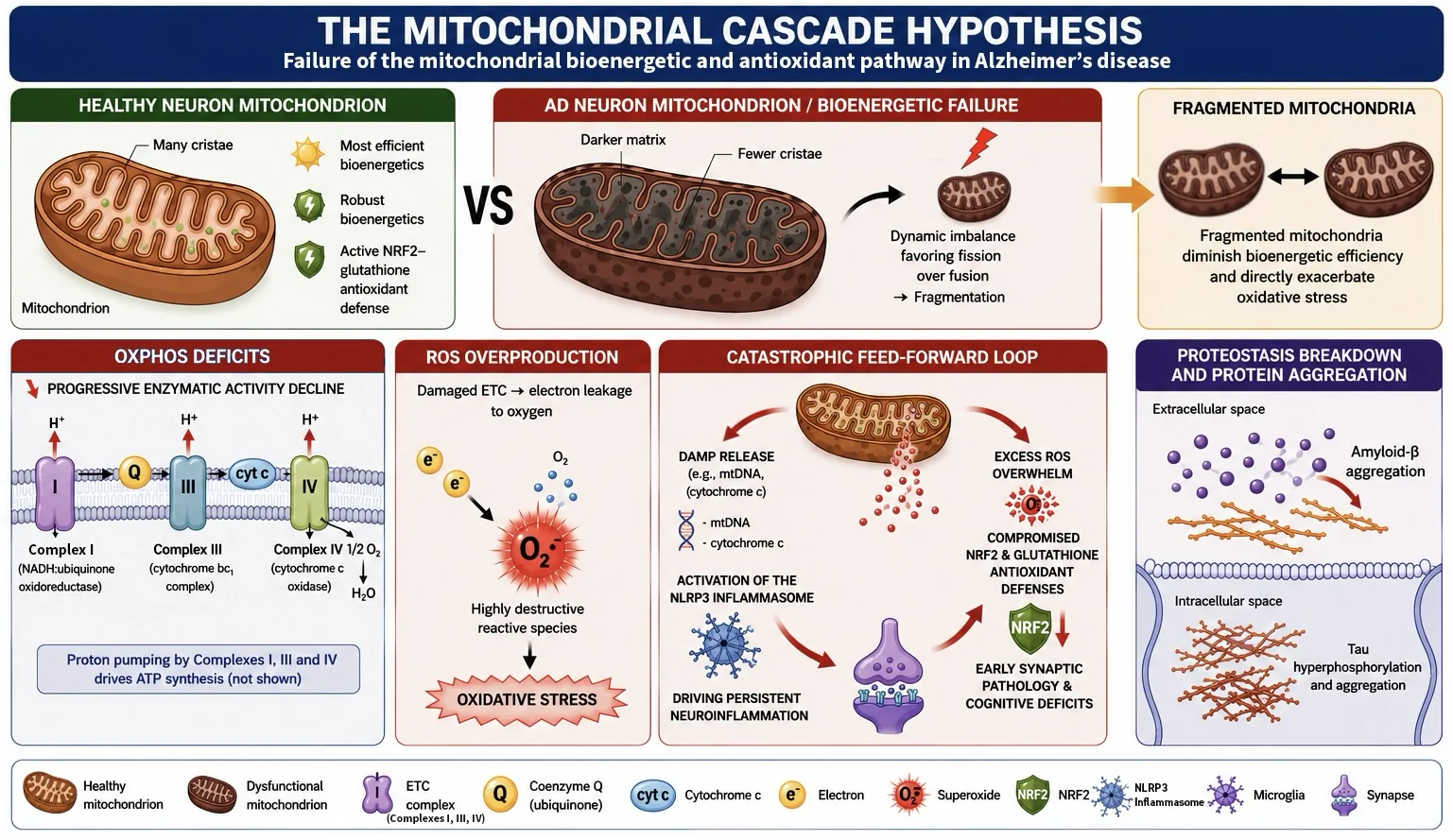
The Mitochondrial Cascade Hypothesis and Bioenergetic Dysfunction in AD. This schematic illustrates the proposed contribution of mitochondrial dysfunction to AD pathogenesis. Healthy neuronal mitochondria maintain efficient oxidative phosphorylation (OXPHOS), balanced mitochondrial dynamics, and effective antioxidant defences. In AD, structural alterations including mitochondrial fragmentation, cristae disruption, and reduced respiratory chain activity impair ATP production and increase ROS generation. Excessive ROS can disrupt neuronal homeostasis and promote oxidative damage and may stimulate the release of mitochondrial damage-associated molecular patterns (DAMPs), including mitochondrial DNA and cytochrome c, which activate innate immune pathways such as the NLRP3 inflammasome. Bidirectional interactions among mitochondrial dysfunction, oxidative stress, amyloid pathology, tau pathology, and neuroinflammation contribute to progressive synaptic dysfunction and neuronal vulnerability.

Ref. [10] Figure 3 summarises the principal mechanisms connecting mitochondrial dysfunction to bioenergetic decline and oxidative stress. The model illustrates how alterations in mitochondrial dynamics, impaired oxidative phosphorylation, and increased ROS generation interact to compromise neuronal metabolism. It further highlights the bidirectional relationships among mitochondrial dysfunction, neuroinflammatory signalling, and protein aggregation that may contribute to progressive disease propagation.

### 3.3. Lipid Dysregulation, Brain Lipidomics, and Loss of Metabolic Inflexibility

As glucose metabolism becomes less efficient, other metabolic routes become important for preserving energy balance in the brain. Prolonged energetic stress may impair the ability to adapt to new conditions related to the changing substrate availability, leading to metabolic inflexibility [2].

The apolipoprotein E4 gene (APOE4), the major genetic risk factor in late-onset AD, has an important function in the described process [44]. The effects of this factor go beyond its influence on amyloid plaque metabolism, as it modulates lipid transport, lipid droplets, cholesterol regulation, vascular properties, and inflammatory processes. Expression of APOE4 by astrocytes results in lipid droplet changes, which lead to lipid storage that is more prone to oxidative modifications [45].

Microglial cells also show metabolic changes. Changes in the lipid droplet number and composition, alterations in cholesterol transport, and defects in lysosomal function impair phagocytosis. Thus, metabolic abnormalities are linked to neuroinflammation as well [46].

Recent lipidomic studies suggest that disturbances in lipid handling extend beyond individual cell types and affect broader neural networks [47,48]. Alterations in phospholipid composition, cholesterol metabolism, myelin integrity, and membrane remodelling have been observed across multiple stages of AD [44]. Emerging evidence indicates that prolonged energetic stress may contribute to changes in myelin lipid turnover and white matter integrity, potentially generating lipotoxic intermediates that further compromise neuronal function [49]. Together, these findings highlight the importance of lipid metabolism as both a compensatory response to energetic stress and a potential source of pathological vulnerability.

### 3.4. Sex-Specific Vulnerabilities in Brain Metabolic Reprogramming and Redox Defence

Sex-related differences in cerebral metabolism may contribute to the increased prevalence of AD observed in women. Among the factors implicated in this vulnerability, oestrogen has received particular attention because of its regulatory effects on glucose metabolism, mitochondrial function, synaptic plasticity, and antioxidant defence systems [50].

The menopausal transition is associated with substantial metabolic remodelling within the brain. Declining oestrogen levels influence glucose utilisation, mitochondrial efficiency, and neuronal bioenergetics, creating a period of metabolic adaptation during which compensatory mechanisms become increasingly important. While many women successfully undergo this transition without developing neurodegenerative disease, genetic and metabolic risk factors may reduce adaptive capacity [51].

Recent longitudinal studies suggest that APOE4 may amplify menopause-associated metabolic changes. Female APOE4 carriers exhibit greater alterations in amino acid metabolism, tricarboxylic acid cycle intermediates, and lipid homeostasis than non-carriers, indicating a reduced capacity to maintain bioenergetic stability during ageing [52].

Oestrogen withdrawal may also influence redox regulation. Experimental evidence suggests that ooestrogen contributes to mitochondrial efficiency and supports endogenous antioxidant defences through both genomic and non-genomic mechanisms. Consequently, the combined effects of oestrogen decline, impaired lipid metabolism, and mitochondrial dysfunction may increase susceptibility to oxidative stress and neuroinflammatory signalling. These interactions may help explain sex-specific differences in disease risk and progression, although further longitudinal studies are required to establish causal relationships [50].

**Figure 4:**
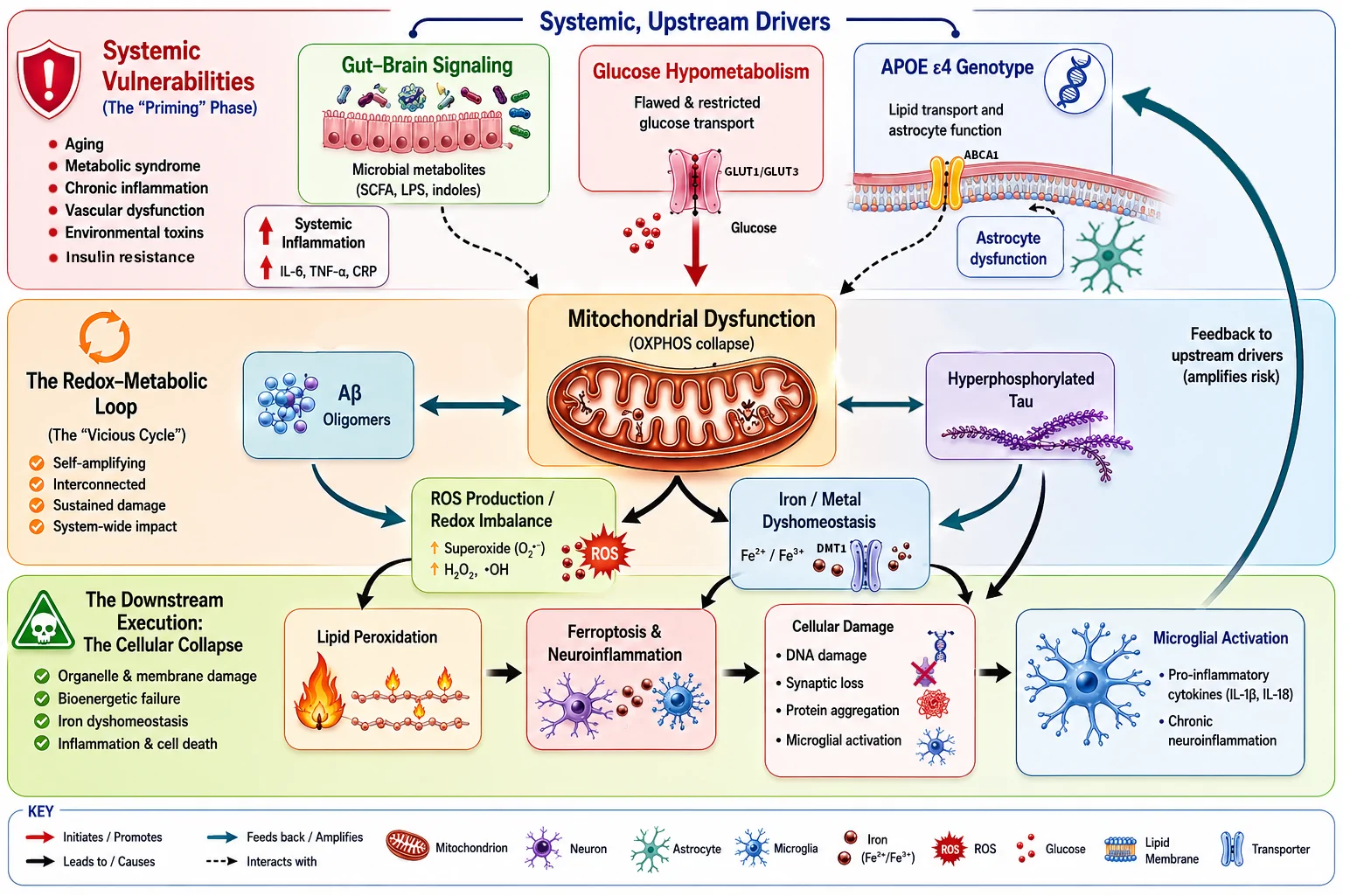
The Redox-Metabolic Network of AD. This schematic integrates major upstream and downstream mechanisms implicated in AD. Systemic vulnerabilities—including glucose hypometabolism, insulin resistance, APOE4-associated lipid dysregulation, altered gut–brain signalling, and age-related loss of metabolic resilience—create conditions that favour mitochondrial dysfunction and oxidative stress. At the centre of the model, reciprocal interactions between impaired oxidative phosphorylation and reactive oxygen species generation establish a self-amplifying redox-metabolic loop. These disturbances promote amyloid and tau pathology, neuroinflammation, transition-metal dyshomeostasis, and lipid peroxidation. The resulting network increases susceptibility to regulated cell death pathways, including ferroptosis, ultimately contributing to synaptic dysfunction, neuronal loss, and cognitive decline. Circular arrows denote reinforcing feedback mechanisms that may accelerate disease progression.

Figure 4 summarises the integration of these metabolic, genetic, and redox-related processes. The schematic presents a systems-level model in which impaired glucose metabolism, mitochondrial dysfunction, APOE4-associated lipid dysregulation, neuroinflammation, and oxidative stress converge within a self-reinforcing redox-metabolic network. This framework provides a conceptual bridge between early metabolic vulnerability and the downstream pathogenic mechanisms discussed in subsequent sections.

## 4. The Pathogenic Triad: Proteostasis, Neuroinflammation, and Systemic Crosstalk

While metabolic dysfunction and oxidative stress create conditions that increase neuronal vulnerability, progression of AD involves the interaction of additional pathological processes that amplify and propagate cellular injury. Among these, disturbances in proteostasis, chronic neuroinflammation, and systemic metabolic and immune signalling have emerged as major contributors to disease progression. Rather than operating independently, these mechanisms form an interconnected pathogenic network in which protein aggregation, glial activation, and peripheral inflammatory signals reinforce one another through multiple feedback pathways. Understanding these interactions is essential for explaining how early metabolic disturbances evolve into widespread neurodegeneration [6,7].

### 4.1. Interaction Between Amyloid-Β, Tau Pathology, and Mitochondrial Oxidative Stress

The interaction between classical proteinopathy associated with AD and oxidative stress is now seen as reciprocal. Aβ and tau pathology may interfere with mitochondrial function and contribute to oxidative stress, which in turn may exacerbate protein misfolding, aggregation, and proteotoxicity [6,7].

The interaction between amyloid pathology and metal dyshomeostasis provides an additional link between proteinopathy and redox metabolism. Aβ can interact with transition metals, particularly copper and iron, and these interactions may influence both Aβ aggregation and the local redox environment. Metal–Aβ complexes can participate in redox reactions under appropriate conditions, potentially contributing to local reactive-species generation, while oxidative modification of Aβ and associated proteins may in turn alter their aggregation, interactions, and clearance [53,54]. Thus, amyloid pathology and metal-associated redox disturbances may participate in reciprocal interactions within the broader redox-metabolic network rather than representing independent pathological processes.

This interaction also helps explain why regional metal accumulation should not be interpreted solely as an isolated disturbance of metal metabolism. Metal-associated redox chemistry may intersect with mitochondrial dysfunction, lipid peroxidation, neuroinflammation, and regulated cell death, thereby providing mechanistic links between Aβ pathology and downstream cellular injury [55,56]. However, whether these interactions represent initiating events or downstream amplifiers in human AD remains unresolved.

Specifically, soluble Aβ oligomers are especially damaging to neurons and have been found to bind to mitochondrial proteins such as amyloid-binding alcohol dehydrogenase (ABAD) and cyclophilin D, resulting in mitochondrial damage and an increased risk of permeability transition pore opening. This impairs mitochondrial membrane potential, affects ATP synthesis, and increases the formation of ROS [57].

Tau-mediated pathology occurs through complementary processes. The hyper-phosphorylated form of tau separates from microtubules and forms helical filaments and neurofibrillary tangles, which disrupt cytoskeletal dynamics and axonal transport. The malfunction in the transport of mitochondria and other organelles prevents the distal parts of the neuron from coping with energy demands and synaptic function [58].

Importantly, oxidative stress may further promote protein aggregation. Elevated ROS levels have been associated with increased expression of β-secretase (BACE1), favouring amyloidogenic processing of amyloid precursor protein (APP). Oxidative stress can also activate kinases involved in tau phosphorylation, including glycogen synthase kinase-3β (GSK3β) and cyclin-dependent kinase 5 (CDK5), thereby facilitating tau pathology. Together, these observations support the existence of reciprocal interactions among mitochondrial dysfunction, oxidative stress, Aβ accumulation, and tau pathology that may contribute to progressive disease propagation [59,60].

### 4.2. Glial Immunometabolism: Microglial Reprogramming and Astrocytic Redox Dysfunction

Microglia and astrocytes play pivotal roles in preserving brain homeostasis under normal conditions. However, in Alzheimer’s disease, continuous exposure to protein aggregates, debris, and inflammatory factors can cause substantial changes in glial metabolic and functional processes. The phenomenon is widely termed immunometabolic reprogramming, which affects glial functions being either adaptive or maladaptive [61].

Microglia exhibit considerable metabolic flexibility; they can shift between oxidative phosphorylation and glycolysis, depending on environmental demands. During early injury responses, increased glycolytic activity may support phagocytosis and tissue repair. However, prolonged activation appears to favour a persistent inflammatory phenotype. This is characterised by increased production of cytokines such as interleukin-1β (IL-1β) and tumour necrosis factor-α (TNF-α) [46]. Disease-associated microglia (DAM) display complex metabolic signatures. These include altered mitochondrial function, impaired lipid handling, and changes in lysosomal activity. All this suggests that chronic activation may eventually compromise cellular homeostasis [61].

Astrocytes undergo analogous changes. Reactive astrocytes are required to maintain glutamate clearance, support neuronal metabolism, regulate blood–brain barrier function, and provide antioxidant protection. However, sustained inflammatory signalling and oxidative stress may impair these homeostatic functions [31]. Experimental studies indicate that disruption of NRF2-dependent transcriptional programmes can reduce glutathione synthesis and weaken antioxidant defences, thereby limiting the capacity of astrocytes to support neighbouring neurons under conditions of metabolic stress [10].

The combined dysfunction of microglia and astrocytes may therefore contribute to a feed-forward cycle in which oxidative stress, inflammatory signalling, and metabolic impairment mutually reinforce one another, accelerating neuronal vulnerability [10,61].

### 4.3. The Gut–Brain Axis: Microbiota-Derived Metabolites and Systemic Inflammation

Recent advances in microbiome research have expanded the conceptual framework of AD beyond the central nervous system, highlighting potential contributions from the gut–brain axis [62,63,64]. Although causal relationships remain under investigation, accumulating evidence suggests that alterations in gut microbial composition may influence neuroinflammation, metabolic regulation, and systemic oxidative stress [65].

In healthy individuals, intestinal microorganisms ferment dietary fibre to produce short-chain fatty acids (SCFAs), including butyrate, propionate, and acetate. These metabolites exert broad physiological effects, including regulating immune responses, maintaining intestinal barrier integrity, and modulating gene expression through epigenetic mechanisms, most notably inhibition of histone deacetylases (HDACs) [66]. Butyrate, in particular, acts as an endogenous HDAC inhibitor and has been shown to influence blood–brain barrier integrity, neuroinflammatory signalling, and synaptic plasticity [67].

Several studies have reported reduced abundance of SCFA-producing bacterial taxa in individuals with AD and mild cognitive impairment [62,65,68]. Although the functional implications remain incompletely understood, diminished SCFA production may contribute to impaired neuroimmune regulation and reduced resilience to inflammatory stressors.

Concurrently, age-related alterations in gut barrier integrity may permit increased translocation of microbial products into the systemic circulation [69]. Lipopolysaccharide (LPS), a component of Gram-negative bacterial cell walls, has attracted particular attention because it can activate innate immune signalling pathways through Toll-like receptor 4 (TLR4). Experimental evidence suggests that circulating inflammatory mediators may influence blood–brain barrier function and promote microglial activation, thereby linking peripheral immune activity with central nervous system inflammation [70].

Although many aspects of gut–brain communication remain under active investigation, current evidence supports the concept that systemic metabolic and inflammatory signals can interact with established AD pathologies. These observations reinforce the view of AD as a multisystem disorder in which peripheral and central processes contribute jointly to disease progression [67,70].

**Figure 5:**
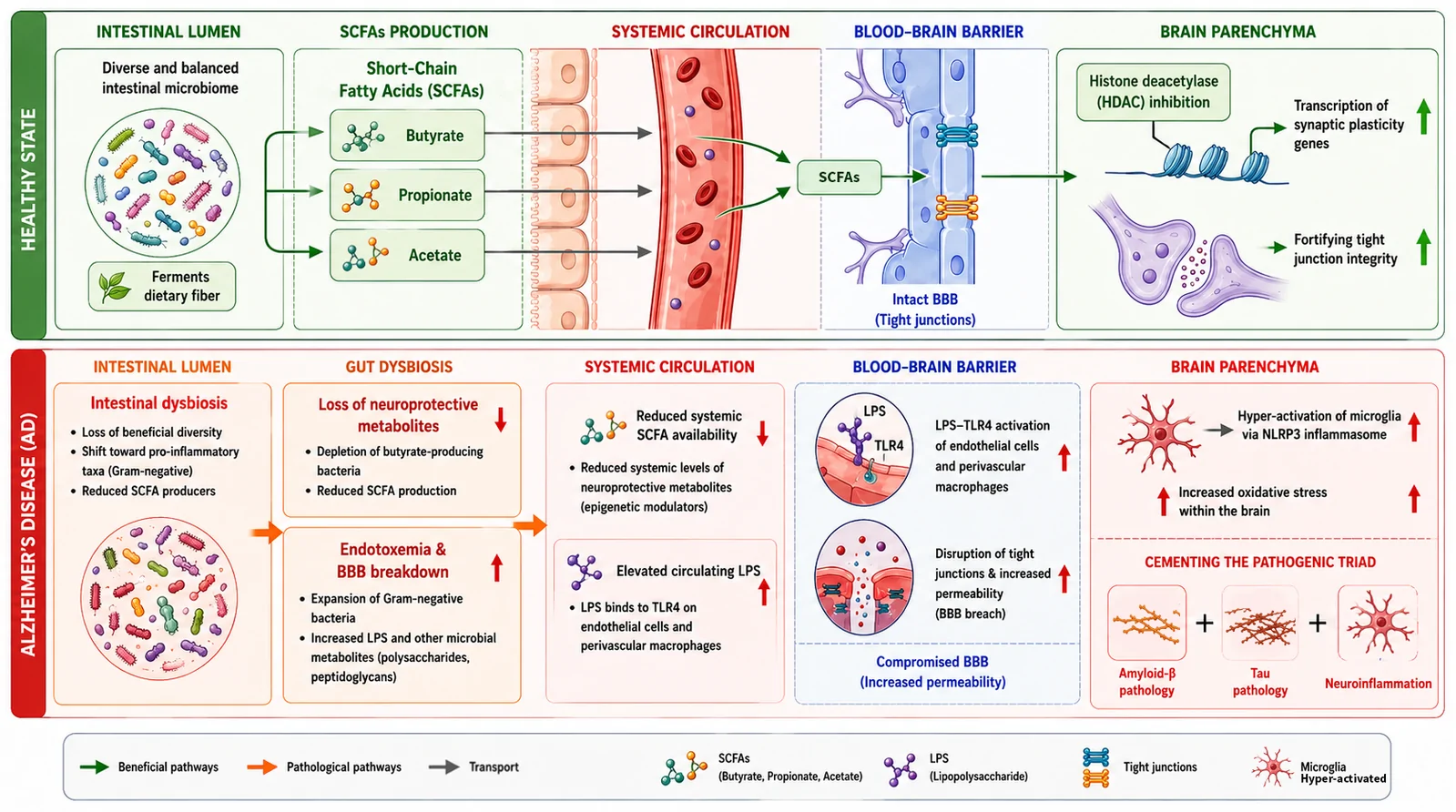
The Gut–Brain Axis and Systemic Crosstalk in AD. This schematic illustrates potential interactions between the intestinal microbiome and the central nervous system in health and AD. In the healthy state, microbial fermentation of dietary fibre generates short-chain fatty acids (SCFAs), including butyrate, propionate, and acetate. These contribute to intestinal barrier integrity, immune regulation, and neuroprotective signalling. In AD, microbial dysbiosis may reduce SCFA production while increasing pro-inflammatory microbial products, e.g., lipopolysaccharide (LPS). Age-related impairment of intestinal and blood–brain barrier function may facilitate systemic dissemination of inflammatory mediators. This promotes microglial activation and neuroinflammatory signalling. Together, these pathways illustrate how peripheral metabolic and immune processes may interact with central mechanisms of oxidative stress, protein aggregation, and neurodegeneration.

Figure 5 summarises the interactions between intestinal microbial ecology, systemic inflammation, blood–brain barrier integrity, and neuroinflammatory signalling. The figure illustrates how alterations in gut microbial composition may influence the production of neuroactive metabolites and inflammatory mediators, thereby linking peripheral physiological processes with central mechanisms of neurodegeneration. This framework highlights the growing recognition of AD as a disorder influenced by both brain-intrinsic and systemic biological networks.

## 5. Metal Dyshomeostasis and Regulated Oxidative Cell Death

Increased evidence exists that neuronal death in AD is caused not only by degeneration but also by the activation of regulated cell death (RCD). Although traditional neuropathology pointed to Aβ deposits and tau neurofibrillary tangles, new data show that metabolic dysregulation, oxidative stress, and metal dysregulation can be crucial factors in cell death [71,72].

Among the factors implicated in this process, iron and copper have received particular attention because of their dual physiological and pathological roles. Under normal conditions, these metals are essential for neurotransmitter synthesis, mitochondrial respiration, myelin maintenance, and antioxidant enzyme function [73]. However, disruption of metal transport, storage, and buffering systems during ageing and neurodegeneration may increase the availability of redox-active metal ions. Thus, this may promote oxidative damage and activate multiple cell death pathways [71,73].

Consequently, metal dyshomeostasis is increasingly viewed as a convergence point linking mitochondrial dysfunction, oxidative stress, neuroinflammation, and regulated cell death. Understanding these interactions may provide novel opportunities for therapeutic intervention [71,74,75].

### 5.1. Iron Accumulation, Lipid Peroxidation, and Ferroptosis

Iron is the most abundant transition metal in the central nervous system and is essential for numerous biological processes, including mitochondrial respiration, haem synthesis, iron–sulphur (Fe–S) cluster formation, and the activity of several metabolic and antioxidant enzymes [76]. Neuroimaging studies employing quantitative susceptibility mapping (QSM) and related magnetic resonance imaging techniques have reported increased iron accumulation in several brain regions affected by AD, including cortical and hippocampal structures [77]. However, increased regional or total tissue iron should not be interpreted as a direct measure of the chemically reactive iron pool. Brain iron is distributed among several functionally distinct compartments, including haem, Fe–S clusters, ferritin-bound iron, and a relatively small labile iron pool (LIP) containing readily exchangeable iron [78]. Consequently, changes in total iron concentration and expansion of the redox-active LIP represent related but distinct phenomena.

Brain iron homeostasis is maintained through coordinated uptake, intracellular trafficking, utilisation, storage, recycling, and export. Circulating transferrin-bound iron is primarily internalised through transferrin receptor 1 (TfR1), followed by endosomal iron release and intracellular trafficking involving divalent metal transporter 1 (DMT1). Iron can subsequently be incorporated into haem and Fe–S clusters, stored within ferritin, or exported through ferroportin. Hepcidin provides an additional regulatory mechanism by promoting ferroportin internalisation and degradation, thereby restricting cellular iron export [79].

These pathways generate several chemically and biologically distinct iron pools, including haem iron, Fe–S cluster iron, ferritin-bound iron, and the relatively small labile iron pool (LIP), which contains readily exchangeable iron capable of participating in redox reactions. Consequently, increased total tissue iron does not necessarily indicate a proportional expansion of the LIP. This distinction is particularly relevant when interpreting neuroimaging findings: quantitative susceptibility mapping (QSM) can detect regional differences in magnetic susceptibility associated with iron-containing compounds, but QSM measurements should not be equated directly with the concentration of redox-active intracellular Fe^2+^ [78,80].

Disruption of these processes during ageing or neurodegeneration may alter intracellular iron distribution and increase the availability of redox-active iron without necessarily producing a proportional increase in total tissue iron.

Similarly, ferritinophagy mediated by nuclear receptor coactivator 4 (NCOA4) can mobilise ferritin-associated iron and potentially increase the LIP under conditions of excessive iron release [80].

When redox-active iron becomes available, Fe^2+^ can participate in radical-generating reactions, including Fenton chemistry:

Fe^2+^ + H_2_O_2_ → Fe^3+^ + •OH + OH^−^

Such reactions can contribute to oxidative modification of proteins, nucleic acids, and membrane lipids [73]. However, ferroptosis should not be equated with nonspecific Fenton-mediated oxidative damage. Rather, ferroptosis is a regulated form of cell death characterised by the iron-dependent accumulation of oxidised phospholipids and phospholipid hydroperoxides, resulting in progressive membrane dysfunction in the absence of the classical molecular features of apoptosis [63]. Its execution depends on the convergence of iron availability, membrane lipid composition, lipid-remodelling pathways, lipid-peroxidation reactions, and the capacity of multiple antioxidant systems to remove oxidised phospholipids.

Particularly important is the susceptibility of neuronal membranes to lipid peroxidation because of their high content of polyunsaturated fatty acids (PUFAs), which provide readily oxidisable substrates [71]. Acyl-CoA synthetase long-chain family member 4 (ACSL4) promotes the incorporation of PUFA-derived acyl chains into membrane phospholipids, thereby increasing the availability of substrates susceptible to peroxidation. Lysophosphatidylcholine acyltransferase 3 (LPCAT3) contributes to the remodelling of these lipids into phospholipid species that can undergo enzymatic and non-enzymatic oxidation. Lipid peroxyl radicals generated during these reactions can propagate chain reactions within PUFA-containing phospholipids, leading to the accumulation of phospholipid hydroperoxides. When their formation exceeds the capacity of cellular detoxification systems, membrane integrity and function may progressively deteriorate [81].

A central defence against this process is the glutathione (GSH)-dependent glutathione peroxidase 4 (GPX4) system, which reduces phospholipid hydroperoxides to the corresponding lipid alcohols and thereby limits lipid-peroxidation-driven membrane damage [82]. The availability of GSH depends in part on cysteine supply through the cystine/glutamate antiporter system Xc^−^. Impairment of system Xc^−^, depletion of GSH, or loss of GPX4 activity can therefore increase susceptibility to ferroptotic injury [69]. However, GPX4 is not the only cellular defence against phospholipid peroxidation. The ferroptosis suppressor protein 1 (FSP1)–coenzyme Q10 (CoQ10) system provides a complementary, membrane-associated antioxidant pathway that limits lipid-radical propagation. Within mitochondria, dihydroorotate dehydrogenase (DHODH) can also help maintain reduced CoQ and thereby protect mitochondrial membranes from lipid peroxidation [83]. These parallel systems indicate that ferroptotic susceptibility depends not on a single antioxidant pathway but on the integrated capacity of cells to maintain phospholipid redox homeostasis.

Iron metabolism further influences this balance by regulating the amount and localisation of redox-active iron available to participate in lipid-peroxidation reactions. Alterations in iron uptake, storage, export, or ferritin turnover may increase the LIP and thereby favour ferroptotic conditions [84]. Nevertheless, increased iron availability alone is insufficient to establish ferroptosis. The cellular consequences depend on the availability of oxidisable phospholipids, the activity of lipid-remodelling enzymes, the rate of lipid-peroxidation reactions, and the efficiency of GPX4- and GPX4-independent antioxidant systems. This distinction is particularly relevant in AD, where iron accumulation, mitochondrial dysfunction, altered lipid composition, and impaired antioxidant capacity may coexist and potentially reinforce one another [56].

Experimental evidence increasingly supports an association between ferroptotic mechanisms and AD-related neurodegeneration. Alterations in ACSL4, GPX4/GSH homeostasis, system Xc^−^ activity, iron handling, and lipid-peroxidation pathways have each been implicated in AD-relevant experimental systems [56,71,73,82,85]. However, the strength of evidence differs among individual mechanisms, and the precise contribution of ferroptosis to human AD remains under investigation. Current findings therefore support ferroptosis as a potentially important mechanism linking disturbances in iron homeostasis, membrane lipid oxidation, and neuronal vulnerability, rather than establishing it as a single or primary cause of neuronal loss [85].

Age-related cellular senescence may further influence this process. Senescent glial and vascular cells can acquire pro-inflammatory phenotypes and display alterations in iron-handling proteins, potentially contributing to changes in local iron homeostasis and the inflammatory environment [86]. Such alterations may increase the susceptibility of neighbouring cells to oxidative and lipid-peroxidation stress. Thus, ageing-associated changes in iron trafficking, lipid metabolism, mitochondrial function, inflammation, and antioxidant capacity may converge to modify ferroptotic vulnerability in AD-relevant brain regions.

Importantly, QSM-derived increases in regional magnetic susceptibility should not be interpreted as direct evidence of an expanded LIP or of ferroptosis. QSM provides information about magnetic susceptibility associated with iron-containing compounds and therefore offers valuable information about regional iron distribution, but it cannot by itself distinguish ferritin-bound iron, haem iron, Fe–S-associated iron, and redox-active intracellular Fe^2+^. Establishing a direct relationship between regional iron accumulation and ferroptotic neuronal death therefore requires complementary biochemical, cellular, and pathological evidence [56].

Overall, ferroptosis provides a mechanistic link between iron dyshomeostasis and membrane lipid damage within the broader redox-metabolic framework of AD. Its relevance is best understood as the result of interactions among iron trafficking, PUFA-containing phospholipid remodelling, lipid-peroxidation chemistry, and multiple antioxidant defence systems. This perspective also emphasises that therapeutic strategies aimed at ferroptosis should not simply remove iron or broadly suppress oxidative reactions. Instead, potential interventions should correct pathological iron availability or lipid-peroxidation processes while preserving essential iron-dependent metabolism and physiological redox signalling. At present, ferroptosis-targeted approaches remain predominantly experimental and require further validation in appropriate cellular, animal, and human AD models.

### 5.2. Cuproptosis: Copper-Dependent Mitochondrial Stress

Copper is an essential transition metal in the brain, where it participates in mitochondrial respiration, neurotransmitter metabolism, antioxidant defence, and numerous enzymatic processes. However, disrupted copper homeostasis may contribute to neuronal dysfunction by altering its distribution, intracellular trafficking, and redox activity. Changes in copper availability can affect mitochondrial function and promote oxidative stress, particularly when the balance between copper-dependent physiological processes and redox-active copper pools is disturbed [75].

Copper dyshomeostasis should be distinguished from cuproptosis. Altered copper distribution, impaired copper trafficking, changes in labile copper pools, or altered copper availability for physiological enzymes do not, by themselves, demonstrate activation of cuproptosis. Copper is required for essential processes, including mitochondrial respiration, neurotransmitter metabolism, and antioxidant defence, and disturbances in its homeostasis may therefore have multiple consequences independent of cuproptotic cell death [74].

Cuproptosis is a novel regulated cell death pathway that is mechanistically distinct from apoptosis, necroptosis, and ferroptosis. Unlike ferroptosis, cuproptosis does not primarily involve lipid peroxidation. Instead, it is triggered by excessive intracellular copper interactions with lipoylated proteins of the tricarboxylic acid (TCA) cycle [74]. A 2025 synthesis of this pathway in AD reinforces that, while brain copper measurements remain inconsistent across studies, redistribution toward elevated labile copper appears more consistently linked to oxidative damage and disease progression than total copper content, highlighting cuproptosis-related proteins as candidate therapeutic targets [75].

Experimental studies indicate that copper binding promotes aggregation of lipoylated mitochondrial proteins, particularly components of the pyruvate dehydrogenase complex, leading to proteotoxic stress and disruption of mitochondrial metabolism [74]. Additional effects include destabilisation of iron-sulfur cluster proteins and impairment of oxidative phosphorylation [74].

Consistent with this, evidence directly linking cuproptosis to neuronal death in AD remains preliminary. Most mechanistic insights derive from cellular and experimental models, and the extent to which cuproptosis contributes to human neurodegeneration requires further investigation. Nevertheless, the discovery of this pathway highlights the broader concept that disturbances in metal homeostasis may influence neuronal survival through multiple convergent mechanisms [75].

### 5.3. Zinc Homeostasis and Its Interaction with AD Pathology

Zinc is an essential metal in the brain, where it contributes to synaptic signalling, enzyme activity, protein structure, transcriptional regulation, and neuronal excitability. Unlike iron and copper, Zn^2+^ is not redox-active under physiological conditions and therefore should not be interpreted primarily through Fenton-type chemistry. Nevertheless, alterations in Zn^2+^ availability can influence Aβ aggregation, synaptic function, metalloprotein activity, and interactions with other transition metals [87,88].

Synaptic zinc is tightly regulated, and abnormal extracellular or intracellular Zn^2+^ concentrations may modify Aβ aggregation and interfere with metal-binding sites on proteins. Zinc also interacts functionally with copper and iron homeostasis, creating potential competition or redistribution among metal-binding proteins and transport pathways. Consequently, zinc dysregulation may contribute to AD pathophysiology through altered protein interactions, synaptic dysfunction, and disturbance of broader metal homeostasis rather than through direct redox cycling [75,87,89].

As with iron and copper, the distinction between altered regional zinc concentrations and disruption of the biologically active zinc pool is important. The therapeutic challenge is therefore not simply to reduce zinc concentrations but to restore appropriate compartmentalisation and availability while preserving its essential physiological functions.

Table 1 summarises the principal physiological functions, homeostatic mechanisms, relevant chemical pools, AD-associated alterations, and potential therapeutic implications of iron, copper, and zinc.

### 5.4. Crosstalk Among Ferroptosis, Necroptosis, Pyroptosis, and Panoptotic Signalling

Neurons and glial cells in the degenerating brain are unlikely to undergo cell death through a single, isolated mechanism. Increasing evidence indicates that several regulated cell-death (RCD) pathways can be activated within the same pathological environment and may interact through shared upstream signals, including oxidative stress, mitochondrial dysfunction, metabolic impairment, inflammatory signalling, and metal dyshomeostasis [92,93]. However, these pathways should not be considered equally established in AD. The available evidence differs substantially by pathway, experimental system, cell type, and extent of validation in human tissue. Accordingly, their potential contribution to AD should be considered within an evidence-graded framework rather than as equivalent mechanisms.

Necroptosis is a regulated lytic form of cell death mediated primarily by the receptor-interacting protein kinases RIPK1 and RIPK3 and the terminal effector mixed lineage kinase domain-like protein (MLKL) [92,94]. Activation of the RIPK1–RIPK3 axis promotes MLKL phosphorylation and oligomerisation, ultimately disrupting plasma-membrane integrity. Oxidative and inflammatory stress can facilitate activation of this pathway, while membrane disruption and the subsequent release of intracellular damage-associated molecular patterns (DAMPs) may further amplify local inflammatory responses. Importantly, experimental studies have identified activation of the RIPK1–RIPK3–MLKL pathway in vulnerable neuronal populations in human AD brain tissue and in experimental AD models [92]. These observations provide evidence that necroptotic signalling is associated with neuronal injury in AD, although the extent to which it represents a primary driver, a downstream response, or a context-dependent component of neurodegeneration remains unresolved.

Pyroptosis represents another regulated form of lytic cell death, characterised by inflammasome activation, inflammatory caspase activity, and formation of plasma-membrane pores by gasdermin proteins. In AD, particular attention has focused on the NLRP3 inflammasome, which can respond to cellular stress signals associated with amyloid-β, mitochondrial dysfunction, oxidative stress, and DAMPs. Activation of NLRP3 can promote caspase-1-dependent processing of interleukin-1β and interleukin-18 and cleavage of gasdermin D (GSDMD), thereby linking inflammatory signalling with membrane damage and cell death [93]. Pyroptotic mechanisms may therefore provide a molecular connection between chronic neuroinflammation and cellular injury. Nevertheless, as with necroptosis, the relative contribution of pyroptosis to neuronal versus glial pathology, and its temporal relationship with other pathological processes in human AD, remain incompletely defined.

Ferroptosis differs mechanistically from both necroptosis and pyroptosis because its defining biochemical feature is the iron-dependent accumulation of oxidised phospholipids and phospholipid hydroperoxides. As discussed above, ferroptotic susceptibility depends on the interaction among iron availability, PUFA-containing phospholipid remodelling, lipid-peroxidation reactions, and antioxidant systems including GPX4/GSH. Experimental and pathological evidence supports an association between ferroptotic mechanisms and AD-related neuronal vulnerability [71,73,82,85]. However, oxidative damage or increased iron concentrations alone are insufficient to establish ferroptosis. Demonstrating ferroptotic cell death requires evidence consistent with its characteristic biochemical machinery and dependence on lipid peroxidation. Thus, ferroptosis currently has substantial experimental support in AD, but its precise causal contribution to human disease progression remains under investigation.

Cuproptosis should be considered separately from both copper dyshomeostasis and ferroptosis. Copper is an essential component of mitochondrial respiration and several antioxidant and metabolic enzymes, and altered copper distribution has been reported in AD. Cuproptosis, however, refers to a specific regulated cell-death mechanism associated with excessive intracellular copper and its interaction with lipoylated mitochondrial proteins, particularly proteins associated with the tricarboxylic acid (TCA) cycle [74,75]. Copper-dependent aggregation of lipoylated proteins, together with destabilisation of Fe–S cluster proteins and disruption of mitochondrial metabolism, can produce proteotoxic and metabolic stress [74]. These molecular features should not be inferred solely from altered copper concentrations. Evidence directly establishing cuproptosis as a mechanism of neuronal death in human AD remains limited, with most available evidence derived from experimental systems and mechanistic studies [75]. Cuproptosis should therefore be regarded as a predominantly experimental mechanism whose relevance to human AD requires further validation.

The distinction between metal dyshomeostasis and metal-dependent RCD pathways is particularly important within the redox-metabolic framework. Altered iron or copper homeostasis can influence mitochondrial function, oxidative chemistry, lipid peroxidation, inflammatory signalling, and cellular metabolism without necessarily activating ferroptosis or cuproptosis. Conversely, disruption of metal handling may increase cellular susceptibility to specific RCD pathways by modifying the availability of redox-active metals or altering mitochondrial and antioxidant functions. Thus, metal dyshomeostasis should be considered an upstream or interacting condition that can influence several forms of cellular injury rather than being treated as synonymous with any individual RCD mechanism [95].

These pathways may also interact functionally. Oxidative and mitochondrial stress can lower the threshold for ferroptotic injury while simultaneously promoting inflammatory signalling. Ferroptosis-associated lipid peroxidation and membrane damage may generate DAMPs that activate immune responses, whereas necroptotic or pyroptotic membrane disruption can further increase extracellular inflammatory signals. In turn, inflammatory cytokines, mitochondrial dysfunction, and metabolic stress may alter iron handling, antioxidant capacity, and lipid metabolism, potentially increasing susceptibility to ferroptotic injury [53]. These interactions provide a plausible mechanistic basis for crosstalk among RCD pathways, but mechanistic convergence should not be interpreted as evidence that these pathways are activated simultaneously or contribute equally to neuronal loss at every stage or in every cell type of AD.

The concept of PANoptosis has emerged to describe coordinated interactions among apoptosis, pyroptosis, and necroptosis through shared molecular signalling platforms [92,93]. This framework may help explain how inflammatory and cell-death signals converge rather than remaining confined to a single canonical pathway. In AD, potential interactions involving inflammasome signalling, caspase regulation, and RIPK1/RIPK3-dependent pathways have been proposed, and a recent AD-focused review has suggested that PANoptotic signalling could represent a convergence point for amyloid, tau, and neuroinflammatory mechanisms [93]. Nevertheless, direct evidence demonstrating PANoptosis as an integrated cell-death mechanism in human AD is currently insufficient. PANoptosis should therefore be presented as an emerging conceptual framework rather than as a clinically established mechanism of AD neurodegeneration.

Taken together, current evidence supports a model in which multiple RCD pathways may operate within a common pathological environment characterised by metabolic dysfunction, mitochondrial impairment, redox dysregulation, inflammation, and metal dyshomeostasis. The strength of evidence, however, is not uniform. Ferroptosis has substantial experimental support and increasing evidence from human AD tissue; necroptotic and pyroptotic signalling have also been detected in human tissue and experimental models; cuproptosis remains predominantly supported by experimental evidence; and PANoptosis represents an emerging framework whose specific contribution to human AD has yet to be demonstrated. These distinctions matter when interpreting the therapeutic implications of RCD pathways and assessing whether a particular mechanism is a potential therapeutic target, a downstream consequence of neuronal stress, or both.

**Figure 6:**
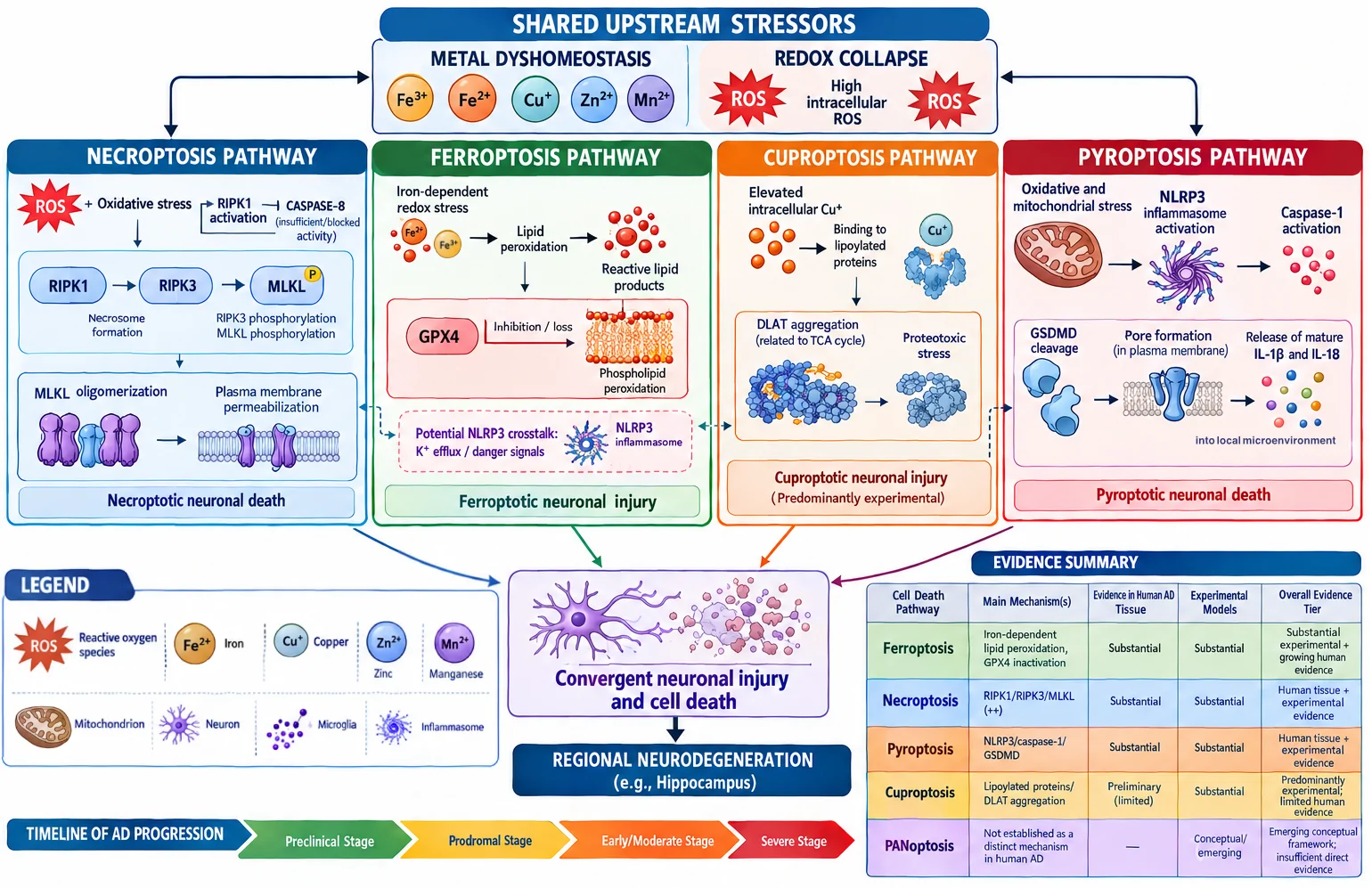
Metal Dyshomeostasis and Integrated Networks of Regulated Cell Death in Alzheimer’s Disease. This schematic illustrates major regulated cell-death pathways discussed in AD and their potential relationships with metal dyshomeostasis, oxidative stress, mitochondrial dysfunction, and inflammatory signalling. Ferroptosis is presented as an experimentally supported mechanism associated with iron-dependent phospholipid peroxidation, whereas cuproptosis remains predominantly experimental and its relevance to human AD has not yet been established. Necroptotic and pyroptotic signalling have been reported in human AD tissue and experimental models, while PANoptotic signalling remains an emerging conceptual framework with insufficient direct evidence in human AD. The figure distinguishes shared upstream stress signals from pathway-specific molecular mechanisms and illustrates proposed areas of mechanistic crosstalk. Directional arrows indicate potential or proposed relationships and should not be interpreted as evidence of obligatory temporal ordering, equivalent causal strength, or equivalent clinical support among the individual pathways. The evidence summary differentiates the current level of support for each regulated cell-death mechanism in human AD and experimental models.

Accordingly, Figure 6 presents these pathways as interconnected mechanisms within the redox-metabolic environment of AD rather than as equivalent or independently established causes of neuronal death. The arrows indicate proposed mechanistic crosstalk and shared pathological conditions, not a fixed sequence of events or equivalent causal evidence. This framework is consistent with the broader view that neuronal degeneration in AD may emerge from reciprocal interactions among proteinopathy, metabolic dysfunction, mitochondrial impairment, redox imbalance, inflammation, metal dyshomeostasis, and several forms of regulated cell death.

## 6. Translating the Redox-Metabolic Framework: Biomarkers and Therapeutic Opportunities

The limited clinical success of therapies directed exclusively at amyloid-beta pathology has encouraged broader exploration of the biological processes that precede overt neurodegeneration in AD [44]. Increasing evidence suggests that metabolic dysfunction, oxidative stress, neuroinflammation, and metal dyshomeostasis emerge early in the disease course and may contribute substantially to disease progression [2]. Consequently, the redox-metabolic framework provides an alternative translational perspective that emphasises early detection of bioenergetic dysfunction and targeted intervention before irreversible neuronal loss occurs.

Rather than replacing established biomarkers, this approach seeks to integrate molecular, metabolic, imaging, and genomic information into a multidimensional precision-medicine strategy. This will identify individuals at risk during the prodromal stages of disease.

### 6.1. Precision Profiling: Multi-Omics and Emerging Redox Biomarkers

The biological heterogeneity of AD presents major challenges for diagnosis, prognosis, and therapeutic selection. Traditional cerebrospinal fluid (CSF) and blood-based biomarkers focused on amyloid-beta and tau provide valuable information regarding pathological burden, but they do not fully capture metabolic dysfunction, oxidative stress, or neuroimmune alterations [96].

Recent advances in single-cell RNA sequencing, spatial transcriptomics, proteomics, metabolomics, and lipidomics have enabled increasingly detailed characterisation of the cellular microenvironment in neurodegenerative disorders. These technologies have revealed substantial heterogeneity among neuronal, astrocytic, microglial, and vascular cell populations, including the identification of disease-associated microglia (DAM), reactive astrocyte subtypes, and region-specific inflammatory signatures [61].

Spatially resolved approaches are particularly valuable because they allow investigators to link molecular signatures with specific anatomical regions exhibiting metabolic dysfunction, oxidative stress, or neuroinflammation. Such analyses may ultimately help identify mechanistically defined patient subgroups and improve therapeutic targeting. A 2024 methodological review of spatial multi-omics platforms in AD compares the resolution, throughput, and analytical trade-offs of current spatial transcriptomic and proteomic technologies, concluding that combining spatial resolution with single-cell profiling is likely necessary to fully resolve microenvironment-specific redox and metabolic alterations around plaques [97].

Complementing tissue-based approaches, blood-derived biomarkers offer the potential for scalable clinical implementation. Emerging lipidomic studies have identified associations between AD and circulating oxidised lipid species, including hydroxyeicosatetraenoic acids (HETEs), oxidised phospholipids (such as oxidised phosphatidylethanolamines (oxPEs), and other markers of lipid peroxidation. Although these biomarkers remain investigational, they may provide insight into systemic oxidative stress and ferroptosis-related vulnerability [98].

Similarly, metabolomic profiling has identified alterations in amino acid metabolism, tricarboxylic acid (TCA) cycle intermediates, redox cofactors, and glutathione metabolism in individuals at risk for cognitive decline. Future studies will determine whether integrating these markers with imaging and genomic data can improve prediction of disease onset and progression [99].

**Figure 7:**
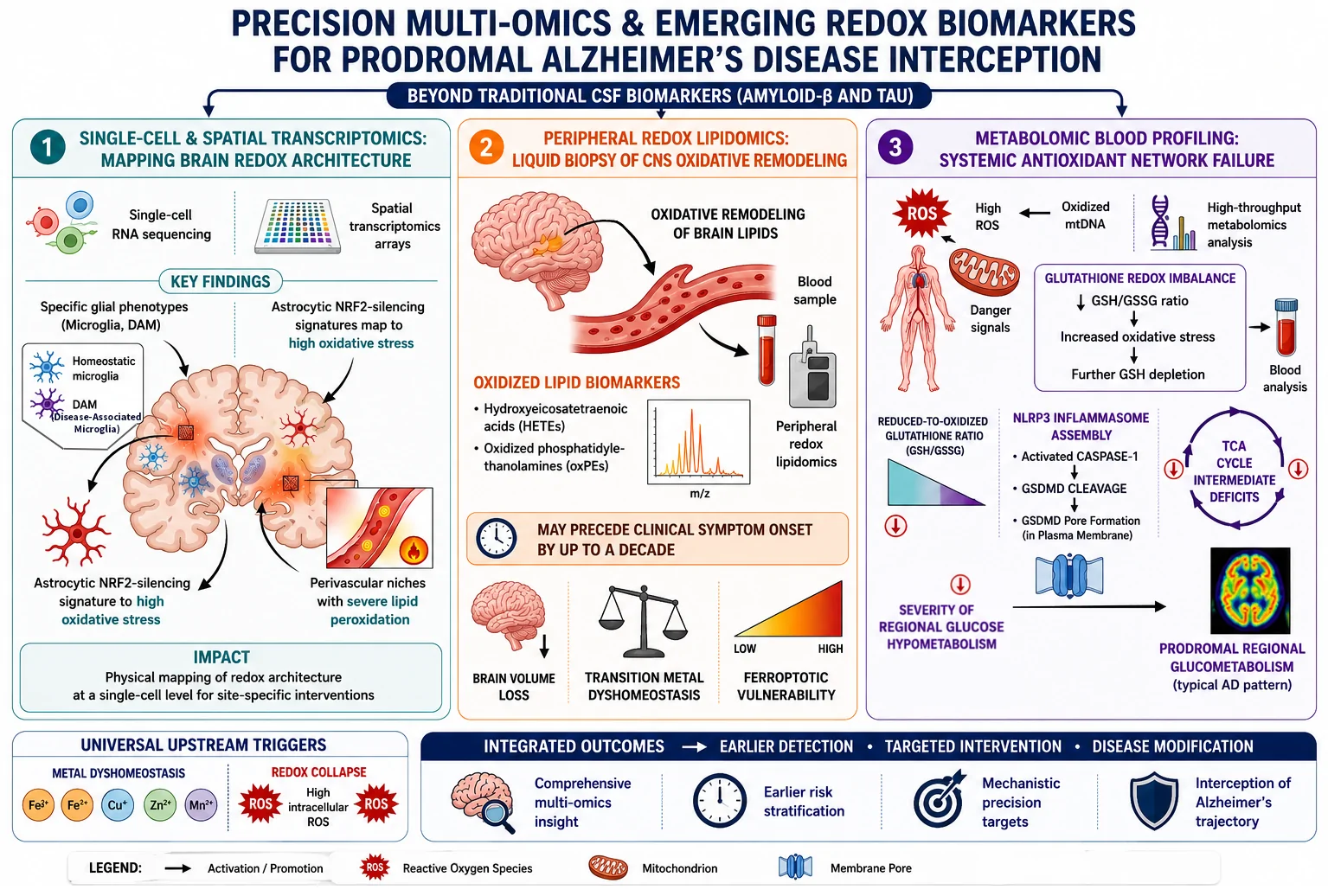
Precision Profiling and Redox-Metabolic Biomarkers for Early AD Detection and Therapeutic Stratification. This schematic illustrates how emerging multi-omics technologies can be integrated with metabolic and imaging biomarkers to improve early detection and patient stratification in AD. Single-cell and spatial transcriptomic approaches identify region-specific cellular responses associated with oxidative stress, neuroinflammation, and metabolic dysfunction. Peripheral lipidomic and metabolomic analyses provide minimally invasive measures of systemic redox status, lipid peroxidation, mitochondrial metabolism, and antioxidant capacity. These data can be combined with established imaging modalities, including FDG-PET, and further stratified by genetic risk factors to generate individualised biological profiles. The resulting framework supports the identification of mechanistically distinct disease subtypes and highlights potential therapeutic targets involving mitochondrial function, NRF2 signalling, metabolic resilience, and regulated oxidative cell death.

The emerging biomarker and therapeutic field derived from the redox-metabolic model of AD is summarised in Figure 7. The figure integrates advances in multi-omics technologies, blood-based metabolic profiling, and systems biology approaches that aim to identify disease-associated alterations before the onset of overt cognitive impairment. Together, these developments support a precision-medicine framework in which biomarker-guided risk stratification may enable earlier, more targeted interventions.

### 6.2. Lessons from Antioxidant Trials: Toward Mitochondrial and Nrf2-Targeted Strategies

Oxidative stress has long been considered a potential therapeutic target in AD. Early intervention strategies largely relied on dietary antioxidants, including vitamin E and vitamin C, based on observational evidence linking antioxidant intake with reduced cognitive decline [8].

However, randomised clinical trials generally failed to demonstrate substantial disease-modifying effects [100]. Several explanations have been proposed. First, oxidative stress in AD is spatially compartmentalised and closely linked to mitochondrial dysfunction, limiting the effectiveness of non-targeted antioxidant supplementation [19,20]. Second, many interventions were administered after significant neurodegeneration had already occurred. Third, oxidative stress may represent one component of a broader pathological network involving metabolic dysfunction, inflammation, and proteostatic failure [8].

These limitations have stimulated interest in strategies that target upstream mechanisms rather than simply scavenging ROS. One approach involves mitochondria-directed compounds: (i) MitoQ, SkQ1 (lipophilic quinone derivatives conjugated to a triphenylphosphonium (TPP^+^) cation, which is selectively accumulated within the inner mitochondrial membrane to prevent lipid peroxidation); (ii) elamipretide (SS-31), a cardiolipin-stabilising peptide, which is designed to accumulate within mitochondria or stabilise mitochondrial membrane architecture [101]. Experimental studies suggest these compounds may improve mitochondrial efficiency and reduce oxidative damage; however, definitive clinical efficacy remains to be established. Preclinical evidence indicates that elamipretide can enhance mitochondrial biogenesis and fusion while reducing oxidative stress and neuroinflammatory markers in models of neurodegeneration, supporting its investigation as a mitochondria-targeted candidate for neurodegenerative disease. However, AD-specific clinical efficacy has not yet been established [102,103].

A complementary strategy focuses on activation of the nuclear factor erythroid 2-related factor 2 (NRF2) pathway. NRF2 regulates a broad transcriptional programme involved in antioxidant defence, glutathione metabolism, iron homeostasis, and cellular stress responses [10]. Pharmacological NRF2 activators such as dimethyl fumarate and omaveloxolone have demonstrated cytoprotective effects in other neurological conditions; thus, these are being investigated for their potential relevance to neurodegenerative diseases [104,105].

Together, these approaches represent a shift from direct free-radical scavenging toward restoration of endogenous cellular defence systems.

### 6.3. Metabolic Interventions and Emerging Cell-Death-Targeted Therapies

Importantly, mechanistic plausibility and therapeutic validation represent distinct levels of evidence. The presence of a potentially druggable pathway in AD does not establish that modifying that pathway will alter clinical disease progression. Accordingly, the therapeutic strategies discussed below are considered according to their biological rationale, experimental evidence, and degree of clinical validation.

Because cerebral glucose hypometabolism is among the earliest detectable abnormalities in AD, considerable interest has focused on therapies that improve metabolic resilience or provide alternative energy substrates [2].

Ketone-based interventions have attracted attention because ketone utilisation remains relatively preserved despite declining glucose metabolism. Medium-chain triglycerides and exogenous ketone formulations increase circulating beta-hydroxybutyrate (β-HB), which can serve as an alternative fuel for neuronal metabolism [2]. Beyond its energetic role, β-HB also functions as an endogenous inhibitor of class I histone deacetylases (HDACs) [106], thereby influencing inflammatory and epigenetic pathways; this HDAC-inhibitory activity has been linked to suppression of NLRP3 inflammasome activation, suggesting potential pleiotropic effects on brain function [107].

Another promising area involves targeting brain insulin resistance. Increasing evidence links impaired insulin signalling with synaptic dysfunction, altered glucose utilisation, and neurodegeneration [17]. Consequently, glucagon-like peptide-1 (GLP-1) receptor agonists—including liraglutide and semaglutide—have been investigated for their neuroprotective potential, motivated by their anti-inflammatory and insulin-sensitising properties. Results from large randomised trials reported in late 2025 have been mixed: the phase 2b ELAD trial of liraglutide did not significantly affect cerebral glucose metabolism but showed a slower decline in executive function and reduced brain volume loss [108], whereas the phase 3 EVOKE and EVOKE+ trials of oral semaglutide did not meet their primary endpoint of slowing clinical progression in early symptomatic AD, despite favourable effects on some AD-related biomarkers [109]. These findings suggest that GLP-1 receptor agonists may offer modest, target-specific benefits rather than broad disease modification, and that further trials in earlier disease stages may be needed to clarify their therapeutic potential.

Anti-amyloid disease-modifying therapies provide important evidence that proteinopathy remains therapeutically relevant. Lecanemab, a monoclonal antibody targeting soluble aggregated Aβ species, produced a statistically significant reduction in clinical decline compared with placebo in the phase 3 Clarity AD trial, although the magnitude of benefit was moderate and treatment was associated with amyloid-related imaging abnormalities and other adverse events [110]. Donanemab similarly reduced cognitive and functional decline in the phase 3 TRAILBLAZER-ALZ 2 trial, with treatment effects varying according to disease stage and tau burden, and subsequently received regulatory approval [111]. These findings argue against interpreting metabolic and redox mechanisms as replacements for amyloid-directed therapy. Instead, they support a broader model in which proteinopathy, metabolic dysfunction, mitochondrial impairment, oxidative stress, and neuroinflammation interact and may provide complementary therapeutic targets. Importantly, the AHEAD 3–45 prevention study is evaluating lecanemab in cognitively unimpaired individuals with preclinical or early preclinical amyloid pathology, directly testing whether amyloid-directed intervention during an earlier stage of the disease continuum can delay subsequent cognitive and pathological progression [112].

The growing recognition of ferroptosis and metal dyshomeostasis has also stimulated interest in therapies targeting regulated oxidative cell death. Experimental approaches include iron-modulating therapies, radical-trapping antioxidants, and compounds that enhance GPX4 activity or glutathione availability. While these interventions remain largely preclinical, they illustrate how mechanistic insights into oxidative cell death could inform future therapeutic development [113].

Synthesising these findings, these approaches emphasise restoration of metabolic competence and cellular resilience rather than targeting a single downstream pathological feature.

Importantly, these approaches differ substantially in their level of clinical validation. Anti-amyloid monoclonal antibodies currently provide the strongest evidence for disease modification in early symptomatic AD, whereas metabolic interventions such as ketone-based strategies and GLP-1 receptor agonists remain investigational, and most ferroptosis-, metal-, mitochondria-, and NRF2-directed approaches remain at preclinical or early translational stages.

### 6.4. Patient Stratification and the Critical Window for Intervention

One of the most important implications of the redox-metabolic framework is the recognition that therapeutic timing may be as important as therapeutic target selection. Numerous studies indicate that metabolic abnormalities, oxidative stress, and inflammatory alterations emerge years before the onset of clinically detectable cognitive impairment [3,114,115].

This observation supports the concept of a critical intervention window during the preclinical and prodromal stages of disease. During this period, neuronal networks remain largely intact and may retain the capacity to respond to metabolic and neuroprotective interventions [3].

Precision medicine approaches will likely require stratification based on genetic, metabolic, and demographic risk factors. Apolipoprotein E (APOE) genotype remains the strongest genetic determinant of late-onset AD risk and influences lipid metabolism, neuroinflammatory responses, and bioenergetic function [44]. Similarly, increasing attention is being directed toward sex-specific differences in disease vulnerability, particularly the metabolic transition associated with menopause and oestrogen decline [51].

Future clinical trial designs may therefore benefit from combining genomic information, blood-based biomarkers, advanced neuroimaging, and metabolic profiling to identify biologically defined patient subgroups. Such approaches could enable earlier intervention and facilitate rational combination therapies that simultaneously target metabolic dysfunction, oxidative stress, inflammation, and regulated cell death pathways.

The translational framework proposed in this review suggests that the greatest therapeutic opportunities may arise not from late-stage reversal of established neurodegeneration, but from early preservation of neuronal metabolic resilience before irreversible structural damage has occurred.

## 7. Limitations, Controversies, and Future Directions

The redox-metabolic framework provides a useful systems-level perspective for understanding AD. Nevertheless, several important conceptual, methodological, and translational challenges remain unresolved. Growing evidence links metabolic dysfunction, oxidative stress, neuroinflammation, and metal dyshomeostasis with disease progression. However, the precise hierarchical relationships among these processes and classical proteinopathies remain actively debated [8,17].

Furthermore, translating mechanistic knowledge into clinically applicable biomarkers and therapies requires overcoming considerable analytical and biological complexity. These limitations should not be seen as weaknesses of the framework itself, but as critical areas for future research.

### 7.1. The Question of Primary Causality: Cause, Consequence, or Reciprocal Interaction?

One central unresolved question in AD research concerns the temporal relationship between metabolic dysfunction and classic pathological features, such as Aβ and tau aggregation.

The traditional amyloid cascade model proposes that the accumulation of toxic Aβ species initiates a descending sequence of mitochondrial dysfunction, oxidative stress, tau pathology, neuroinflammation, and neuronal loss. Within this framework, metabolic alterations are traditionally considered secondary events to the proteinopathy [44].

Conversely, the redox-metabolic model highlights evidence indicating that cerebral glucose hypometabolism, mitochondrial dysfunction, and oxidative stress can be detected years or even decades before the onset of clinical symptoms. These observations raise the possibility that bioenergetic failure may contribute to the initiation or acceleration of amyloidogenic and tau protein-related processes [3].

However, increasing evidence suggests that these opposing perspectives may not be mutually exclusive [116]. Amyloid accumulation, tau pathology, mitochondrial dysfunction, oxidative stress, and neuroinflammation appear to influence each other through multiple feedback mechanisms. Consequently, the challenge may not be to identify a single triggering event, but rather to understand how interacting pathological processes reinforce each other during disease progression.

Future longitudinal studies integrating molecular imaging, blood biomarkers, and multi-omics approaches will be essential to clarify the temporal sequence of these events and identify the first viable therapeutic targets. Accordingly, the redox-metabolic framework should be regarded as a mechanistic, testable model of reciprocal disease amplification rather than definitive evidence that metabolic or redox dysfunction is the single initiating event in AD.

### 7.2. The Redox Paradox and the Challenge of Therapeutic Specificity

A major challenge for redox-based therapies is derived from the dual biological role of ROS and RNS. Excessive oxidative stress contributes to cell damage. However, physiological levels of reactive species are essential for normal cell signalling, synaptic plasticity, immune regulation, and vascular function [8].

This phenomenon, often referred to as the “redox paradox,” complicates therapeutic intervention. Excessive ROS suppression can interfere with normal signalling pathways. But insufficient suppression may fail to prevent oxidative damage. The disappointing results of many trials with conventional antioxidants may partly reflect this challenge [117].

Consequently, effective redox therapies are likely to require greater spatial and temporal precision than previous approaches. Compounds targeting mitochondria aim to reduce oxidative stress at major ROS production sites. This action should also preserve physiological signalling elsewhere. Similarly, modulating endogenous antioxidant pathways such as NRF2 can offer advantages over indiscriminate free radical scavenging by restoring adaptive cellular responses rather than completely eliminating reactive species [10]. However, excessive or chronic NRF2 activation can also have adverse consequences. This underscores the need for context-appropriate dosing rather than indiscriminate activation [118].

Further complexity arises from cell-type-specific differences in antioxidant regulation. Neurons, astrocytes, microglia, and endothelial cells have distinct metabolic requirements and redox regulatory systems. Consequently, interventions that are beneficial in one cellular compartment may produce undesirable effects in another. This fact highlights the need for increasingly sophisticated targeted therapeutic strategies [27,31].

### 7.3. Biomarker Interpretation and the Challenges of Peripheral Measurements

The discovery of minimally invasive biomarkers is an important goal of precision medicine for AD. Yet the correlation between systemic biomarkers and pathological processes in the brain is not always straightforward.

Many redox-metabolic markers have been suggested (oxidised lipids, amino acid metabolites, glutathione markers, inflammatory factors, and molecules secreted by microorganisms), but their changes could reflect other systemic factors unrelated to neurodegeneration. For example, obesity, type 2 diabetes, cardiovascular diseases, chronic inflammation, and ageing affect circulating metabolic signatures [89].

Moreover, blood–brain barrier integrity varies between patients and across disease stages; therefore, it affects the correlation between concentrations of the same biomarker in the brain and peripheral circulation. It makes the analysis of biomarkers dynamic very difficult and could decrease the specificity of the test [119]. The combination of redox-metabolic biomarkers and traditional plasma-based markers (p-tau217, glial fibrillary acidic protein, neurofilament light chain, and Aβ42/40 ratio) is probably needed [96].

Modern techniques like spatial transcriptomics, single-cell sequencing, and multiplex imaging have significantly advanced our understanding of diseased cell states. Nonetheless, these are still mainly used for research purposes and cannot be applied in daily clinical settings due to technical limitations. Hence, translating discoveries into diagnostics represents a major challenge [97].

### 7.4. Biological Heterogeneity and the Concept of a Therapeutic Window

Another major challenge is the significant biological heterogeneity among AD patients. Genetic background, sex, age, vascular health, metabolic status, and environmental exposure all influence the trajectory of disease progression and treatment response [120].

The apolipoprotein E ε4 (APOE4) allele is a prime example of this complexity. Compared to non-carriers, APOE4 carriers exhibit significant alterations in lipid metabolism, neuroinflammatory responses, blood–brain barrier integrity, and bioenergetic function [44]. Therefore, treatment strategies effective in one subgroup may be ineffective in another.

Similarly, sex-specific biological factors may also influence disease susceptibility and progression. Menopausal transition is associated with significant changes in brain glucose metabolism, mitochondrial function, and systemic endocrine signalling. This suggests that women and men may have different critical susceptibility periods [51].

These observations suggest that successful intervention depends not only on selecting appropriate therapeutic targets but also on identifying the optimal stage of intervention. Therefore, future research must determine whether a biologically significant treatment threshold exists (sometimes informally referred to as the “irreversible point”). Beyond this threshold, the efficacy of metabolic rescue strategies likely diminishes significantly.

Ultimately, understanding bioheterogeneity reinforces the need for individualised risk assessments, biomarker-guided patient stratification, and precision medicine approaches in clinical trials and future treatment implementation.

Table 2 summarises the key concepts, methods, and translational challenges related to the redox-metabolic framework. In conclusion, these unresolved issues highlight the complexity of AD biology and underscore the need for longitudinal and multimodal studies. These should integrate the metabolic, inflammatory, genetic, and protein homeostasis dimensions of AD progression.

Importantly, most of these limitations do not argue against a redox-metabolic contribution to AD; rather, they emphasise that AD is likely best understood as a complex systems disorder in which metabolic dysfunction, proteostasis failure, neuroinflammation, and cell-death signalling evolve through highly interconnected and context-dependent feedback networks. Future research should therefore focus on identifying the temporal hierarchy and therapeutic vulnerability of these interacting pathways rather than seeking a single universal initiating event [8,17].

## 8. Conclusions and Future Directions

Integrating the Redox-Metabolic Framework into Precision Neurology.

AD remains one of the most complex and challenging disorders in modern medicine. Although the amyloid cascade hypothesis has profoundly shaped our understanding of AD pathology, growing evidence suggests that neurodegeneration cannot be fully explained by protein aggregation alone [2,116]. Throughout this review, we have examined converging data indicating that mitochondrial dysfunction, cerebral glucose hypometabolism, oxidative stress, neuroinflammation, metal dyshomeostasis, and regulated cell death are intimately interconnected components of disease progression [6,7].

Rather than viewing amyloid-beta and tau pathology as isolated initiating events, the redox-metabolic framework proposes that AD emerges from progressive disturbances in cellular energy homeostasis and redox regulation that interact dynamically with proteostatic and inflammatory pathways. In this model, mitochondrial dysfunction, oxidative stress, and impaired metabolic resilience create a permissive environment that promotes protein aggregation, neuroinflammation, synaptic dysfunction, and ultimately neuronal loss. Importantly, these processes appear to operate through reciprocal feed-forward mechanisms, suggesting that AD is best understood as a systems-level disorder rather than a disease driven by a single pathogenic pathway [6,17].

### 8.1. Oxidative Stress as a Central Integrator of Neurodegenerative Processes

One key point from this literature review is that oxidative stress should not be treated as an indirect consequence of neurodegeneration. Rather, redox dysregulation may function as a systems-level integrator linking mitochondrial dysfunction, metabolic derangement, neuroinflammation, and regulated cell death [10].

Physiologically, ROS play a role in intracellular signalling, synaptic plasticity, and cellular adaptive responses. At the same time, persistent mitochondrial dysfunction may alter ROS production and redox signalling, particularly when antioxidant and repair capacity is insufficient [8]. These changes result in ferroptosis, pyroptosis, necroptosis, and other forms of programmed cell death, which are increasingly considered key mechanisms of AD.

This approach reevaluates oxidative stress not only as an indirect marker of the disease but also as a possible mediator where various pathological processes can converge. Hence, restoring redox balance could be more beneficial than treating a particular process.

### 8.2. Towards Targeted Redox-Metabolic Treatments

The translational implications of the redox-metabolic framework extend from understanding disease mechanisms to identifying therapeutic strategies that may complement existing disease-modifying approaches. However, the current evidence does not support considering redox-metabolic interventions as established treatments for AD. Instead, their therapeutic relevance should be evaluated by the level of clinical evidence, the biological target addressed, and the stage of disease at which intervention is initiated. The failure of conventional antioxidant therapies suggests that nonspecific radical scavenging is unlikely to be sufficient and that more targeted interventions addressing metabolic dysfunction, mitochondrial impairment, endogenous antioxidant defence, or oxidative cell-death pathways may be required [8,10].

Importantly, recent clinical advances in amyloid-directed therapy provide evidence that proteinopathy remains therapeutically relevant. Lecanemab and donanemab have demonstrated a moderate but significant slowing of clinical decline in selected patients with early symptomatic AD, establishing amyloid removal as a clinically validated disease-modifying strategy. These findings should not be interpreted as evidence against metabolic or redox mechanisms. Instead, they support a broader model in which amyloid and tau pathology interact dynamically with mitochondrial dysfunction, metabolic impairment, oxidative stress, and neuroinflammation. Consequently, redox-metabolic interventions may be most appropriately considered as complementary strategies, potentially used alongside established treatments or investigated in biomarker-defined populations and earlier stages of disease. Studies such as AHEAD 3–45, which evaluates anti-amyloid intervention during preclinical AD, further emphasise the importance of therapeutic timing and biological staging.

Within this framework, three major therapeutic domains can be considered. First, mitochondrial protection and restoration of endogenous antioxidant capacity represent mechanistically attractive approaches. Mitochondria-targeted antioxidants and membrane-stabilising compounds, including strategies based on lipophilic cations or Szeto–Schiller peptides such as elamipretide (SS-31), are designed to reduce mitochondrial oxidative damage and preserve bioenergetic function. Similarly, pharmacological activation of NRF2 signalling could enhance endogenous antioxidant and cytoprotective responses [2,10]. These approaches have substantial mechanistic and preclinical support; however, their efficacy as disease-modifying treatments for AD has not yet been established clinically. They should therefore be regarded as investigational rather than established therapeutic interventions.

Second, strategies aimed at restoring metabolic resilience seek to compensate for impaired cerebral glucose utilisation and insulin signalling. Ketone-based interventions may provide an alternative oxidative substrate for neurons and may additionally influence inflammatory and epigenetic signalling pathways. GLP-1 receptor agonists have also attracted considerable interest because of their effects on insulin signalling, metabolism, inflammation, and neuronal function. Nevertheless, clinical evidence remains mixed. The ELAD trial of liraglutide did not significantly improve cerebral glucose metabolism, while the phase 3 EVOKE and EVOKE+ trials of oral semaglutide did not meet their primary endpoint for slowing clinical progression in early symptomatic AD, despite effects on selected disease-related biomarkers. These findings indicate that metabolic interventions remain investigational, and their potential efficacy may depend on disease stage, metabolic phenotype, and appropriate patient selection rather than representing broadly effective disease-modifying therapies.

Third, targeting regulated oxidative cell death and metal dyshomeostasis offers another experimental therapeutic avenue. Ferroptosis-directed strategies, including radical-trapping antioxidants and approaches designed to preserve glutathione-dependent GPX4 activity, may limit lipid-peroxidation-driven neuronal injury. Modulating iron and copper homeostasis is another potential strategy because excessive or mislocalized transition metals can contribute to oxidative damage and mitochondrial dysfunction. However, these approaches remain predominantly preclinical, and their development requires particular caution because iron and copper are essential for normal neuronal and mitochondrial function. The therapeutic objective should therefore be to correct pathological metal accumulation or reactivity without disrupting physiological metal-dependent processes.

These three domains are mechanistically interconnected. Improving mitochondrial efficiency could reduce excessive ROS generation, strengthening endogenous antioxidant systems could increase cellular resistance to oxidative injury, and improving metabolic flexibility could preserve ATP production under conditions of impaired glucose utilisation. At the same time, limiting ferroptotic or metal-dependent damage could prevent oxidative stress from progressing to irreversible neuronal injury. However, mechanistic convergence should not be equated with demonstrated therapeutic synergy, and the efficacy and safety of combined interventions remain to be established experimentally and clinically.

**Figure 8:**
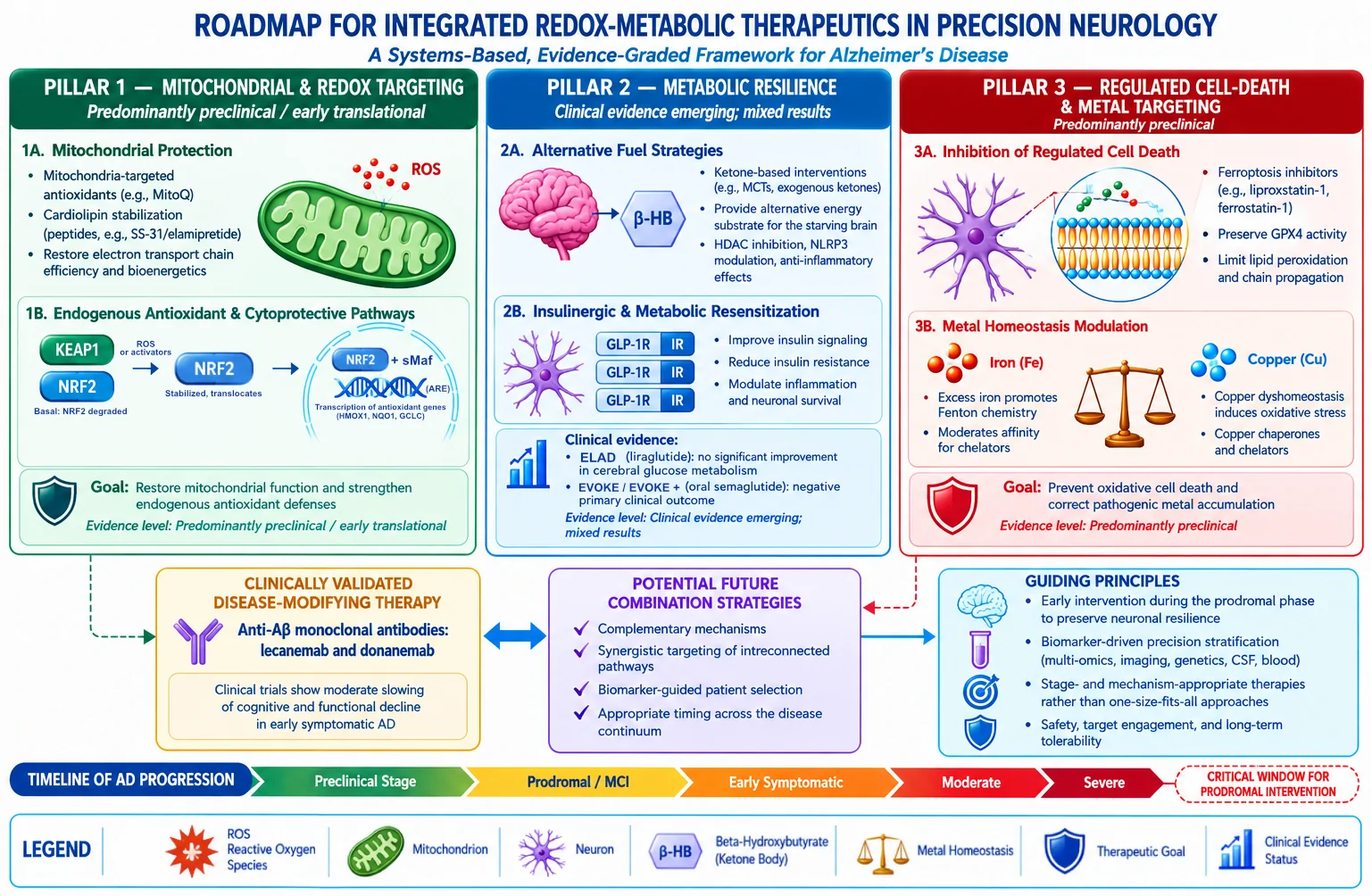
Roadmap of Redox Metabolic Therapeutics for Precision Medicine in AD. This diagram presents a systems-based, evidence-graded framework for investigating therapeutic strategies targeting redox and metabolic mechanisms in Alzheimer’s disease (AD). Three investigational therapeutic pillars are presented: (1) mitochondrial and redox targeting, including mitochondria-targeted antioxidants, cardiolipin-stabilising strategies, and NRF2 activation, which remain predominantly supported by preclinical and early translational evidence; (2) metabolic resilience, including alternative fuel strategies such as ketone-based interventions and insulinergic/metabolic resensitisation with GLP-1 receptor agonists, for which clinical evidence is emerging and remains mixed; and (3) regulated cell-death and metal targeting, including ferroptosis-directed strategies and modulation of iron and copper homeostasis, which remain predominantly preclinical. These three pillars are therefore presented as investigational approaches rather than established treatments. Anti-Aβ monoclonal antibodies, specifically lecanemab and donanemab, are shown separately as clinically validated disease-modifying therapies for appropriately selected patients with early symptomatic AD; clinical trials have demonstrated a moderate slowing of cognitive and functional decline in this population. The figure further illustrates potential future combination strategies in which redox-metabolic interventions could complement established anti-amyloid therapy, although such combinations remain to be validated in appropriately designed clinical trials. The proposed therapeutic framework emphasises biomarker-guided patient stratification, disease staging, preservation of neuronal metabolic resilience, and selection of stage- and mechanism-appropriate interventions, together with appropriate attention to safety, target engagement, and long-term tolerability. The timeline conceptually represents disease progression and the potential prodromal therapeutic window, not a fixed temporal or causal sequence. Overall, the figure distinguishes clinically validated therapy from emerging and predominantly preclinical approaches and should be interpreted as a translational roadmap rather than a clinically validated treatment algorithm.

The therapeutic roadmap presented in Figure 8 should therefore be interpreted as a translational framework rather than a clinically validated treatment algorithm. The three proposed domains differ substantially in their evidentiary status: anti-amyloid monoclonal antibodies currently have the strongest clinical evidence for disease modification in appropriately selected patients with early symptomatic AD; metabolic approaches, including ketone-based interventions and GLP-1 receptor agonists, remain supported by mixed or emerging clinical evidence; and mitochondria-targeted, NRF2-directed, ferroptosis-, and metal-modulating strategies remain largely preclinical or at early translational stages. This distinction is essential to avoid overstating the therapeutic maturity of the redox-metabolic framework.

Equally important, the potential value of these interventions may depend on when and in whom they are administered. Metabolic dysfunction, mitochondrial abnormalities, and redox disturbances may precede substantial neuronal loss, suggesting that the prodromal phase could represent a particularly important period for therapeutic investigation. Biomarker-guided stratification may therefore be essential for determining whether a patient exhibits sufficient metabolic or redox vulnerability to benefit from a particular intervention. In this context, integration of blood-based biomarkers, metabolic imaging, lipidomics, transcriptomics, proteomics, genetic information, and established AD biomarkers could facilitate the identification of biologically defined subgroups and enable more precise evaluation of emerging redox-metabolic therapies [97].

Overall, the redox-metabolic framework provides a rationale for expanding rather than replacing current therapeutic approaches to AD. Its principal translational contribution is not identifying a single alternative treatment target, but recognising that mitochondrial function, metabolic resilience, redox regulation, inflammation, proteostasis, and regulated cell death may constitute interconnected therapeutic dimensions. Future studies should determine whether targeting these processes, alone or in rational combination with established amyloid-directed therapies, can produce clinically meaningful benefits, particularly when interventions are initiated during biologically defined early stages of disease. This approach may ultimately help transform the redox-metabolic framework from a mechanistic model into a testable strategy for precision intervention in AD.

### 8.3. Future Directions

Future research must consider several aspects. First, longitudinal investigations that integrate imaging, metabolomics, lipidomics, genomics, and proteomics are required to identify the temporal connections among metabolic disorders, oxidative stress, protein aggregation, and neuroinflammation. Second, studies must identify whether certain molecular variants of AD have different levels of vulnerability to metabolic and redox disorders. Finally, future clinical trials must include biomarker-driven patient stratification based on various parameters including APOE genotype, gender, metabolism, and disease stage.

In parallel, the greatest opportunity for disease modification may reside within the long prodromal phase of AD, when metabolic dysfunction is already detectable but widespread neuronal loss has not yet occurred [2]. Identifying this therapeutic window and intervening before irreversible neurodegeneration develops will be essential for translating mechanistic insights into clinical benefit.

Overall, the redox-metabolism paradigm is not meant to supplant current models of AD but to embed them in a larger systems biology model that acknowledges the complex interplay between metabolism, oxidative stress, proteostasis, neuroinflammation, and regulated cell death. This paradigm will enable a platform that could support future precision medicine treatments for diagnosing, stratifying risk, and treating patients with AD. Only time will tell if these advancements make a difference in clinical practice, but a new direction for AD research is now outlined.

The evidence reviewed indicates that oxidative stress is unlikely to be adequately described as either the sole triggering mechanism or merely a secondary consequence of AD pathology. Instead, redox dysregulation may function as a systems-level amplifier that links metabolic dysfunction, mitochondrial impairment, proteostasis, neuroinflammation, and regulated cell death throughout disease progression. Redox dysregulation can serve as a systems-level amplifier linking metabolism, proteostasis, neuroinflammation, and programmed cell death throughout all disease stages, an idea that the upcoming longitudinal and intervention studies will be able to address directly.

Figure 8 below schematically presents the translational significance of the redox-metabolic theory, illustrating a possible precision-neurology approach to AD treatment. The proposed strategy combines three therapeutic areas, namely, mitochondrial repair, metabolic assistance, and blocking of regulated oxidative cell death, while paying special attention to the early stage of disease development.

## Figures and Tables

**Table 1:** Iron, copper, and zinc homeostasis in Alzheimer’s disease: physiological functions, molecular regulation, pathological alterations, and therapeutic considerations.

Metal	Major Physiological Functions	Principal Homeostatic Mechanisms/Proteins	Relevant Chemical States/Pools	AD-Associated Alterations	Potential Therapeutic Implications
Fe	Haem synthesis; Fe–S clusters; mitochondrial respiration; enzymatic catalysis	Transferrin/TfR1; DMT1; ferritin; ferroportin; hepcidin; NCOA4	Fe^2+^/Fe^3+^; haem; Fe–S clusters; ferritin-bound iron; labile iron pool	Regional iron accumulation; altered trafficking/storage; possible changes in LIP; increased ferroptotic susceptibility	Correct pathological iron accumulation or reactivity without disrupting essential iron-dependent processes [73,76]
Cu	Cytochrome c oxidase; antioxidant enzymes; neurotransmitter metabolism; mitochondrial function	CTR1; ATP7A/ATP7B; ceruloplasmin; copper chaperones	Cu^+^/Cu^2+^; protein-bound and labile copper pools	Altered distribution and labile copper; interactions with Aβ; oxidative and mitochondrial effects	Restore appropriate copper compartmentalisation/reactivity without disrupting essential cuproenzymes [74,75]
Zn	Synaptic signalling; enzyme function; protein structure; transcriptional regulation; neuronal excitability	ZnT and ZIP transporters; metallothioneins	Zn^2+^; protein-bound and labile synaptic pools	Altered synaptic Zn; effects on Aβ aggregation; altered metal cross-talk	Restore appropriate zinc compartmentalisation and availability rather than indiscriminate depletion [90,91]

**Table 2:** Major Conceptual, Methodological, and Translational Challenges in the Redox-Metabolic Framework of AD.

Challenge	Scientific Basis	Current Evidence Gap	Implications for Research and Clinical Practice
Primary Causality	Mitochondrial dysfunction, oxidative stress, amyloid-β accumulation, and tau pathology interact through reciprocal feed-forward loops [17].	Longitudinal human studies cannot yet definitively establish which pathological process emerges first.	Uncertainty regarding whether interventions should primarily target metabolism, proteostasis, inflammation, or combinations thereof.
The Redox Paradox	Physiological ROS and RNS are essential signalling molecules involved in synaptic plasticity, long-term potentiation, and cellular adaptation [8].	Therapeutic thresholds separating beneficial from pathological redox signalling remain poorly defined.	May explain the failure of broad-spectrum antioxidant trials and necessitate compartment-specific therapies.
Spatial Redox Compartmentalisation	Oxidative stress differs across mitochondria, cytosol, lysosomes, glia, neurons, and vascular cells [19,20].	Most biomarkers and therapies cannot currently distinguish among these distinct redox microenvironments.	Limits precision targeting and may contribute to variable treatment responses.
Peripheral Biomarker Confounding	Circulating lipidomic, metabolomic, and inflammatory markers are influenced by systemic diseases including diabetes, obesity, and cardiovascular disorders [17].	The relationship between blood biomarkers and localised brain pathology remains incompletely characterised.	Potential for false-positive results, inaccurate staging, and reduced diagnostic specificity.
Blood–Brain Barrier Variability	BBB permeability changes with ageing, APOE genotype, vascular dysfunction, and disease progression [44].	Dynamic BBB changes complicate interpretation of circulating biomarkers and therapeutic delivery.	Challenges longitudinal monitoring and precision drug targeting.
Clinical Translation of Multi-Omics	Spatial transcriptomics, single-cell sequencing, and advanced lipidomics provide unprecedented mechanistic resolution [61,97].	Most technologies remain expensive, technically demanding, and restricted to research settings.	Limits widespread implementation in routine clinical practice and large-scale screening programmes.
Patient Heterogeneity	APOE genotype, sex, age, vascular burden, metabolic health, and environmental exposures significantly influence disease trajectories [44].	Current clinical trials often treat AD as a biologically homogeneous disorder.	Necessitates biomarker-guided patient stratification and personalised therapeutic approaches.
Defining the Therapeutic Window	Metabolic dysfunction may precede symptoms by 10–20 years, whereas neuronal loss becomes progressively irreversible [2].	The biological threshold at which metabolic rescue becomes ineffective remains unknown.	Complicates trial design and highlights the importance of prodromal intervention.
Metal Dyshomeostasis Complexity	Iron and copper contribute to oxidative stress, ferroptosis, and cuproptosis, yet are also essential for normal neuronal physiology [71,74].	Safe modulation of cerebral metal pools without disrupting physiological functions remains challenging.	This limits the development of metal-targeted therapies and increases the risk of adverse effects.
Network Redundancy of Cell Death Pathways	Ferroptosis, pyroptosis, necroptosis, and apoptosis exhibit extensive molecular crosstalk through PANoptotic signalling networks [92,93].	It remains unclear which pathway predominates at specific disease stages.	Single-target therapies may prove insufficient, supporting multi-target therapeutic strategies.

## Data Availability

No new data were created or analysed in this study. Data sharing is not applicable to this article.

## Abbreviations

The following abbreviations are used in this manuscript:

ABAD	Amyloid-binding alcohol dehydrogenase
ACSL4	Acyl-CoA synthetase long-chain family member 4
AD	Alzheimer’s disease
AHEAD 3–45	Anti-Amyloid Treatment in Asymptomatic Alzheimer’s Disease (clinical trial)
ANLS	Astrocyte-Neuron Lactate Shuttle
APOE	Apolipoprotein E
APOE4	Apolipoprotein E ε4 allele/gene
APP	Amyloid precursor protein
ARE	Antioxidant response element
ATP	Adenosine triphosphate
ATP7A/ATP7B	Copper-transporting P-type ATPases A/B
Aβ	Amyloid-beta
BACE1	Beta-site Amyloid Precursor Protein Cleaving Enzyme 1
CDK5	Cyclin-dependent kinase 5
CoQ10	Coenzyme Q10
CTR1	Copper transporter 1
CSF	Cerebrospinal fluid
DAM	Disease-associated microglia
DAMPs	Damage-associated molecular patterns
DHODH	Dihydroorotate dehydrogenase
DMT1	Divalent metal transporter 1
ELAD	‘Evaluating Liraglutide in Alzheimer’s Disease’ (clinical trial)
EVOKE/EVOKE+	Phase 3 clinical trials of oral semaglutide in early symptomatic Alzheimer’s disease
FDG-PET	Fluorodeoxyglucose positron emission tomography
FSP1	Ferroptosis suppressor protein 1
GLP-1	Glucagon-like peptide-1
GLUT1	Glucose transporter 1
GLUT3	Glucose transporter 3
GPX4	Glutathione peroxidase 4
GSDMD	Gasdermin D
GSH	Glutathione
GSK3β	Glycogen synthase kinase-3β
HDAC(s)	Histone deacetylase(s)
HETEs	Hydroxyeicosatetraenoic acids
IL-18	Interleukin-18
IL-1β	Interleukin-1β
KEAP1	Kelch-like ECH-associated protein 1
LIP	Labile iron pool
LPCAT3	Lysophosphatidylcholine acyltransferase 3
LPS	Lipopolysaccharide
LTP	Long-term potentiation
MCT1/2/4	Monocarboxylate transporter 1/2/4
MLKL	Mixed lineage kinase domain-like protein
NAD+	Nicotinamide adenine dinucleotide (oxidised form)
NADPH	Nicotinamide adenine dinucleotide phosphate (reduced form)
NCOA4	Nuclear receptor coactivator 4
NLRP3	NOD-, LRR- and pyrin domain-containing protein 3
NMDA	N-methyl-D-aspartate
NO	Nitric oxide
NRF2	Nuclear factor erythroid 2-related factor 2
oxPEs	Oxidised phosphatidylethanolamines
OXPHOS	Oxidative phosphorylation
oxPUFAs	Oxidised polyunsaturated fatty acids
p-tau217	Phosphorylated tau at threonine 217 (blood biomarker)
PANoptosis	Combined apoptosis, pyroptosis, and necroptosis cell death pathway
PI3K/Akt	Phosphoinositide 3-kinase/protein kinase B signalling pathway
QSM	Quantitative susceptibility mapping
RCD	Regulated cell death
RIPK1/3	Receptor-interacting protein kinase 1/3
RNS	Reactive nitrogen species
ROS	Reactive oxygen species
SCFAs	Short-chain fatty acids
SS-31	Elamipretide (mitochondria-targeted, cardiolipin-stabilising peptide)
TCA	Tricarboxylic acid
TfR1	Transferrin receptor 1
TLR4	Toll-like receptor 4
TNF-α	Tumour necrosis factor-α
TPP+	Triphenylphosphonium cation
ZIP	Zrt/Irt-like protein (zinc transporter, SLC39 family)
ZnT	Zinc transporter (SLC30 family)
β-HB	Beta-hydroxybutyrate
